# Liquid Crystalline Self‐Assembly with Accelerated Kinetics and Higher Structural Orderliness in Centrifugal Acceleration Fields Beyond 7251 Times Gravity of Earth

**DOI:** 10.1002/advs.202415955

**Published:** 2025-04-25

**Authors:** Lingyan Xu, Hongbo Zhao, Pei‐Xi Wang

**Affiliations:** ^1^ School of Nano‐Tech and Nano‐Bionics University of Science and Technology of China 96 Jinzhai Road Hefei Anhui 230026 P. R. China; ^2^ i‐Lab Suzhou Institute of Nano‐Tech and Nano‐Bionics of the Chinese Academy of Sciences 398 Ruoshui Road Suzhou Jiangsu 215123 P. R. China

**Keywords:** cellulose nanocrystals, centrifugal acceleration fields, colloidal lyotropic liquid crystals, entropy‐driven self‐assembly, hierarchically ordered structures

## Abstract

Gravity of the Earth (**g**) drives the macroscopic differentiation of multiple phases with different volumetric mass densities in many chemical and physical processes. Herein, liquid crystalline phase separation of colloidal dispersions of rod‐shaped cellulose nanoparticles in centrifugal acceleration fields up to 71 061 meters per second squared (7251 **g**) is studied. Through non‐ionic in situ free‐radical polymerization initiated by time‐controllable oxidation–reduction reactions between tert‐butyl hydroperoxide (oxidants) and thiourea (thiocarbamide, reductants) at room temperature (298 kelvins), ordered soft microstructures formed by entropy‐driven self‐assembly are immobilized within crosslinked polyacrylamide matrixes at various evolution stages (e.g., after 10, 30, or 60 min) in centrifuge tubes. Based on cross‐sectional polarized optical and scanning electron microscopy, strong centrifugal acceleration fields accelerated the movement velocity of discrete liquid crystalline tactoidal microphases, the coalescence of tactoids into continuous chiral nematic structures, as well as the translational and rotational relaxation rates of mesogenic nanorods at kinetically arrested states in high‐viscosity concentrated colloidal liquid crystals, leading to the elimination of topological defects and improvements in structural orderliness. Since acceleration is indistinguishable from a homogeneous gravitational field according to Einstein's principle of equivalence in general relativity, these results might help to predict self‐assembling behaviors near compact astrophysical objects such as neutron stars.

## Graphical abstract

How do chemical and physical processes involving multiple phases develop on compact astrophysical objects such as white dwarf stars? Herein, entropy‐driven self‐assembly of rod‐shaped nanoparticles in centrifugal acceleration fields up to 7251 times the gravity of Earth is investigated, where strong acceleration fields accelerated the kinetics of phase separation and improved the structural orderliness of high‐viscosity colloidal liquid crystalline dispersions.

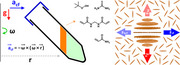

## 1 Introduction

**Figure 1:**
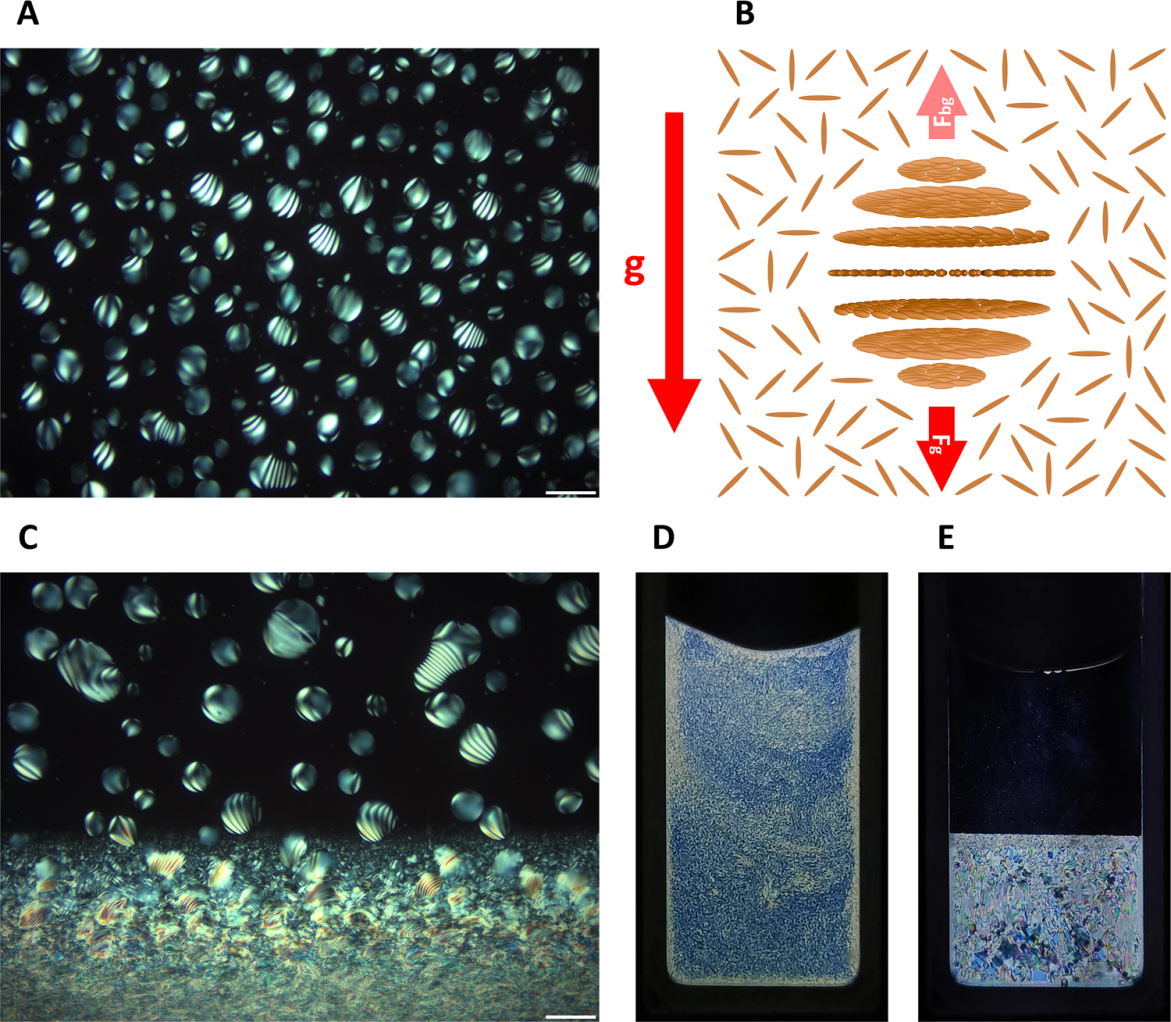
A) A polarized optical microscopy image showing chiral nematic liquid crystalline tactoids in an aqueous dispersion of cellulose nanocrystals (5.6 wt.%). B) In the gravitational acceleration field of the Earth, an individual tactoid is driven by the vector sum of its weight (**F_g_**, vertically downward) and the gravitational buoyancy force (**F_bg_**, vertically upward) from the surrounding isotropic phase. C) A POM image showing the coalescence between tactoids and a continuous anisotropic phase at the bottom of a dispersion. D,E) Photographs of an aqueous dispersion of CNCs (5.6 wt.%, sealed in a vertical glass cuvette with an internal width of 9.84 mm and an optical path length of 1.0 mm) before and after phase separation. In (A), (C), (D), and (E), the samples were placed between two perpendicularly oriented linear polarizers in the North‐South and East‐West directions and illuminated with transmitted white light. Scale bars: (A,C) 100 µm.

Since the pioneering work of Gray in 1992,^[^ ^1^ ^]^ left‐handed chiral nematic liquid crystalline phases formed by the self‐assembly of cellulose nanocrystals (CNCs) in aqueous colloidal dispersions have been widely used to fabricate nanomaterials with helical microstructures,^[^ ^2^, ^3^, ^4^, ^5^, ^6^ ^]^ which showed potential applications in circularly polarized light sources,^[^ ^7^ ^]^ chiral optical devices,^[^ ^8^, ^9^ ^]^ asymmetric separation,^[^ ^10^ ^]^ photonic encryption,^[^ ^11^ ^]^ etc. The self‐assembly processes of CNCs have been found to be mediated by tactoids, which are discrete liquid crystalline microdomains existing in continuous isotropic phases (**Figure** **1**A).^[^ ^12^, ^13^, ^14^ ^]^ CNCs (with an average mass density of ≈1.46 g cm^−3^) in anisotropic phases are able to occupy the space with greater efficiency since these rod‐shaped mesogens are orderly arranged,^[^ ^15^ ^]^ and therefore the mass densities of tactoids are slightly higher (≈0.1–0.2%) than those of disordered phases (Figure 1B).^[^ ^16^, ^17^, ^18^ ^]^ Upon standing, tactoids gradually settle to the bottom of the dispersions as driven by the gravitational acceleration field of the Earth (**g**, ≈9.8 m s^−2^ near the surface of this planet) (Figure 1C), and eventually coalesce into macroscopic chiral nematic structures (Figure 1D,E).^[^ ^19^, ^20^, ^21^ ^]^ This process occurs in many lyotropic liquid crystals and colloidal self‐assembling systems, such as those formed by vanadium pentoxide,^[^ ^22^ ^]^ polypeptides,^[^ ^23^ ^]^ DNA,^[^ ^24^ ^]^ chitin nanocrystals,^[^ ^25^ ^]^ carbon nanotubes,^[^ ^26^ ^]^ graphene oxide,^[^ ^27^ ^]^ chromonic salts,^[^ ^28^ ^]^ amyloid,^[^ ^29^ ^]^ and hydroxyapatite.^[^ ^30^ ^]^ However, as the differences between the mass densities of ordered and disordered phases are usually minimal, the kinetic driving forces for the sedimentation and coalescence of tactoids are very weak, resulting in slow movement velocities of tactoids and long phase‐separation times.^[^ ^31^ ^]^

Although it is difficult to specifically manipulate liquid crystalline tactoids since these fluid anisotropic microdroplets are compositionally almost the same as surrounding isotropic phases, several methods have been developed to influence the movements of tactoids and to further affect phase‐separation processes. By confining CNC dispersions in narrow spaces, e.g., in capillary tubes,^[^ ^32^ ^]^ microspheres,^[^ ^33^ ^]^ or microcavities,^[^ ^34^ ^]^ coalescence between tactoids might complete quickly to give highly integrated chiral nematic phases with low densities of defects. Removal of water from CNC dispersions could be accelerated by vacuum filtration, resulting in iridescent films with improved homogeneity.^[^ ^35^ ^]^ The MacLachlan group reported the differentiation between tactoids and isotropic phases in volume magnetic susceptibility by introducing superparamagnetic doping nanoparticles,^[^ ^36^ ^]^ which enabled kinetic control of the speed and direction of phase separation with weak gradient magnetic fields from permanent magnets. Furthermore, when mixed with polymers exhibiting liquid–liquid phase separation behaviors, the self‐assembly of CNCs may be thermodynamically regulated to realize complex hierarchical structures.^[^ ^37^ ^]^ However, the above strategies are usually not suitable for additive‐free large‐volume CNC dispersions that are most widely used in the fabrication of chiral nematic photonic materials. An important phenomenon that determines the final liquid crystalline structures in CNC suspensions (and many other lyotropic liquid crystals) is the increase in viscosity with the concentration, which eventually slows down the relaxation rates of mesogenic nanoparticles and therefore transforms the colloidal systems into kinetically arrested states.^[^ ^38^, ^39^ ^]^ Once the dispersions have been kinetically arrested, rearrangements of the self‐assembled microstructures and healing of topological defects (formed during the fusion between multiple misaligned tactoids) would become extremely slow, where improvement of orderliness may require significantly prolonged static standing periods, e.g., 15 days.^[^ ^40^ ^]^ Therefore, increasing the intrinsic movement velocities of tactoids and relaxation rates of mesogens are critical for the structural engineering of liquid crystalline phases of cellulose nanocrystals.

**Figure 2:**
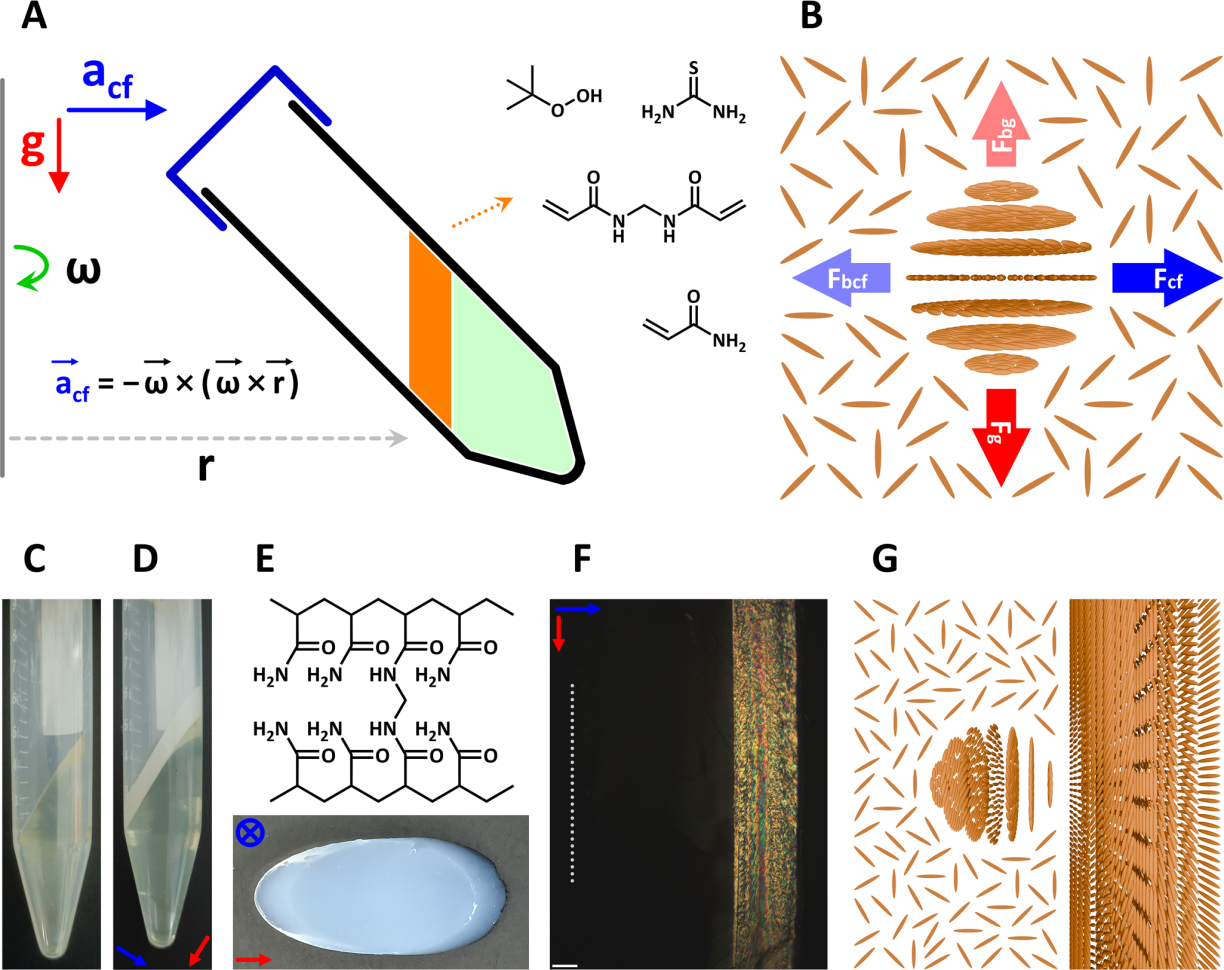
A) The experimental setup for time‐controlled immobilization of self‐assembled microstructures in acceleration fields up to 71061 m s^−2^ generated by a centrifuge. B) In an accelerating reference frame that is rotating with the rotor around its vertically oriented central axis with an angular velocity of **ω**, a liquid crystalline tactoid is driven by the vector sum of its weight (**F**_g_, vertically downward), the fictitious centrifugal force (**F**_cf_, horizontally outward from the rotation axis), the gravitational buoyancy force (**F**_bg_, vertically upward), and the fictitious centrifugal buoyancy force (**F**_bcf_, horizontally inward toward the rotation axis) from the surrounding continuous isotropic phase. C–E) By solidification of liquid epoxy resins (depicted in green in A) inside a centrifuge tube during centrifugation in a fixed‐angle rotor, a plane surface parallel to the rotation axis was formed. An aqueous dispersion (orange‐colored in A) containing CNCs and precursors of polyacrylamide was then added, and the centrifuge tube was sealed and placed back into the rotor with the relative positions between them kept the same. Centrifugation was performed again at 298 K, and polymerization was initiated by redox initiators after a predetermined period of time during centrifugation, rapidly forming a hydrogel that could be further taken out of the centrifuge tube. F) Liquid crystalline structures captured within the crosslinked polyacrylamide matrix were observed by polarized optical microscopy from sliced cross‐sections of the hydrogel. The sample was placed between two crossed polarizers in the North‐South and East‐West directions, and the original air‐liquid interface (before polymerization) is labeled with a dotted line. G) Driven by the centrifugal acceleration fields, tactoids moved horizontally to the right (away from the axis of rotation) and coalesced into a macroscopic chiral nematic phase. Scale bar: (F) 200 µm.

Herein, we investigated the entropy‐driven self‐assembly processes of cellulose nanocrystals and the evolution of lyotropic liquid crystalline phases in centrifugal acceleration fields up to 71 061 meters per second squared (≈7251 times gravity). Based on the time‐controllable redox reactions between tert‐butyl hydroperoxide ((CH_3_)_3_C─O─O─H, oxidants) and thiourea ((H_2_N)_2_C═S, thiocarbamide, reductants) at room temperature (≈298 kelvins), free‐radical polymerization of acrylamide (monomers) and *N*,*N*'‐methylenebis(acrylamide) (crosslinkers) could be initiated at specific time points (e.g., 10, 30, and 60 min after mixing) to rapidly capture and immobilize self‐assembled soft microstructures (such as tactoids) within crosslinked polyacrylamide matrixes in centrifuge tubes (**Figure** **2**A,B). As revealed by optical and electron microscopy, the evolution of liquid crystalline phases at 71 061 m s^−2^ was significantly faster than that at 9.8 m s^−2^, where highly ordered long‐range chiral nematic structures with remarkably lower concentrations of topological defects could be formed in 1 h. Moreover, when subjected to strong centrifugal acceleration fields, kinetically arrested states in concentrated (10% by weight) viscous CNC dispersions were readily overcome. At 71 061 m s^−2^, the translational and rotational relaxation kinetics of cellulose nanocrystals, as well as the reorganization of self‐assembled microstructures were dramatically accelerated, thus transforming the initially chaotic system of numerous misaligned cholesteric microdomains into unidirectionally aligned and periodically spaced parallel chiral nematic layers within 60 min (the term “chiral nematic layers” used here and after refers to the stripes in the periodically “layered” structures revealed by polarized optical microscopy and cross‐sectional scanning electron microscopy when observing a chiral nematic liquid crystalline phase through a direction perpendicular to the helical axis; in each of these “chiral nematic layers”, the orientation of mesogenic cellulose nanocrystals rotates by 180 degrees from one side to the other side. However, the structure of a chiral nematic phase is actually continuous, and there are no physical interfaces to separate it into distinct layers). It should be noted that these misalignment‐to‐alignment reconfiguration processes were not accompanied by the emergence and transformations of chiral nematic tactoids since the dispersions were already completely liquid crystalline (without any isotropic regions) due to their high concentrations, and the gel‐like glassy colloidal liquid crystals behaved similarly to thermotropic liquid crystals consisting of small molecules with intrinsically higher relaxation rates. These results suggest a time‐efficient approach to highly ordered chiral nematic superstructures, and also provide a simple method to investigate the influences of acceleration fields on the entropy‐driven lyotropic liquid crystalline self‐assembly of anisotropic colloidal nanoparticles.

## 2 Immobilization of Liquid Crystalline Microstructures in Centrifugal Acceleration Fields by In Situ Redox‐Initiated Free‐Radical Polymerization at Room Temperature

Cellulose nanocrystals used in this research were prepared by partial hydrolysis of finely ground powders of dry filter paper (5.0 grams) in an aqueous solution of sulfuric acid (64 wt.%, 100 mL) pre‐heated to a temperature range of 318–323 K. The reaction mixture was stirred for 60 min before being quenched with cold water, then solids were collected by centrifugation and ionic species were removed by dialysis against deionized water,^[^ ^41^ ^]^ finally giving a stable colloidal dispersion with an acidic pH of 3.0 and a negative zeta‐potential of −57.2 millivolts. Under transmission electron microscopy (Figure S1, Supporting Information), CNCs appeared as rod‐shaped nanoparticles with lengths of 170–480 nm and widths of 11–26 nm. In a representative experiment, an aqueous dispersion (5.6 wt.%) of the prepared CNCs phase‐separated into an upper isotropic phase with a density of 1.0142 g cm^−3^ and a lower anisotropic phase with a density of 1.0160 g cm^−3^. The MacLachlan group studied the liquid crystalline behaviors of CNCs confined in glass capillary tubes (with internal diameters of ≈0.4 mm) rotated by an overhead stirrer at 500 rpm, where the centrifugal acceleration fields may be weaker than 28 **g** if the distance between the rotation axis and the end of the capillary tube was less than 100 mm, and the chiral nematic structures would also be significantly affected by the spatial confinement besides rotation.^[^ ^42^ ^]^ In order to investigate the effects of strong centrifugal acceleration fields (100 000 m s^−2^ or higher) on the liquid crystalline self‐assembly of cellulose nanocrystals in unconfined bulk colloidal dispersions, our experiments were conducted in centrifuge tubes mounted in a fixed‐angle rotor in a centrifuge machine that could operate at 12 000 rpm or higher speeds, and then the most critical problem was how to quickly immobilize or solidify the self‐assembled soft microstructures formed during centrifugation after a predetermined period of evolution time without mechanical disturbances or vibrations, otherwise, the fluid structures would be severely distorted by the gravity of the Earth or destroyed by shaking once centrifugation was stopped.

To minimize the disruptive influences from geometrical confinements and asymmetrical boundary environments, a flat plane liquid–solid interface perpendicular to the centrifugal acceleration field vectors was designed. A liquid mixture (≈3.4 mL) of epoxy resins and hardeners was added to an empty 15‐mL plastic centrifuge tube (with an internal diameter of 14.66 mm and an external diameter of 16.50 mm), the tube was placed into the fixed‐angle rotor, and centrifugation was performed at 9000 rpm for 35 min, during which time the liquid epoxy resins solidified at the end of the tube to give a plane surface parallel to the rotation axis of the rotor (Figure 2A,C; Figure S2, Supporting Information), where the distance between the plane surface and the rotation axis was 80 millimeters. Afterward, an aqueous dispersion (1.0 mL, deoxygenated by bubbling nitrogen for 60 s) containing cellulose nanocrystals (≈57.6 mg), acrylamide (1.407 mol L^−1^, monomer), *N*,*N*'‐methylenebis(acrylamide) (64.86 mmol L^−1^, crosslinker), and thiourea (6.57 mmol L^−1^, reductant) was homogeneously mixed with tert‐butyl hydroperoxide (30.96 mmol L^−1^, oxidant) and added into the tube. The centrifuge tube was tightly sealed and placed back into the fixed‐angle rotor with the relative positions between them kept the same as previously marked, then centrifugation was immediately performed again at a rotation speed of 9000 revolutions per minute (942.48 radians per second) and a constant temperature of 298 kelvins for 70 min. During centrifugation, free‐radical chain polymerization of acrylamide and *N*,*N*'‐methylenebis(acrylamide) was initiated by the redox reactions between tert‐butyl hydroperoxide and thiourea. A crosslinked polyacrylamide hydrogel matrix was rapidly formed, and the ordered soft microstructures self‐assembled by cellulose nanocrystals were stabilized by in situ immobilization (Figure 2D; Figure S3A–E, Supporting Information). Upon drying, a hydrogel would shrink by ≈25% horizontally and ≈55% vertically (Figure S3F–I; Table S1, Supporting Information). The freshly prepared cellulose‐polyacrylamide composite hydrogel (with a thickness of ≈2.96 mm) showed moderate flexibility and robustness (Figure S4, Supporting Information), which was taken out of the centrifuge tube while maintaining its integrity (Figure 2E), and then sliced with a razor blade to give fresh cross‐sections for examination between two perpendicularly oriented linear polarizers using polarized optical microscopy. An anisotropic layer (≈672 microns in thickness) exhibiting strong birefringence was observed at the bottom of the hydrogel (here the original liquid–solid interface between the unpolymerized dispersion and the solidified epoxy resins was defined as the bottom surface of the hydrogel), indicating the formation of a macroscopic liquid crystalline phase at the farther end away from the rotation axis during centrifugation (Figure 2F). Moreover, above this birefringent layer, the upper part of the hydrogel (which was closer to the rotation axis during centrifugation) displayed almost no birefringence, suggesting that the differentiation of ordered and disordered phases had thoroughly completed before polymerization.

## 3 Influences of Centrifugal Acceleration Fields on Discrete Liquid Crystalline Tactoidal Microphases

Considering a non‐inertial (accelerated) reference frame that is rotating with the fixed‐angle rotor around its vertically oriented central axis with an angular velocity omega **ω**, and in this rotating reference frame, an individual liquid crystalline tactoidal microphase surrounded by a continuous isotropic phase is present at a distance **r** from the rotation axis (Figure 2B). The movement direction and velocity of this tactoid would be mainly influenced by four external forces, which are the weight (**F**_g_, vertically downward) of the tactoid originated from the gravity of the Earth, the gravitational buoyancy force (**F**_bg_, vertically upward) from the surrounding isotropic phase, the fictitious centrifugal force (**F**_cf_, radially outward away from the rotation axis) caused by the rotation of the system, and the fictitious centrifugal buoyancy force (**F**_bcf_, radially inward toward the rotation axis) from the surrounding isotropic phase that is also rotating with the same angular velocity **ω**:

(1) $$dF_{g}=\rho_{\left(\mathrm{LC}\right)}\;g\;dV_{\left(\mathrm{LC}\right)}$$

(2) $$dF_{\mathrm{bg}}=-\rho_{\left(\mathrm{Iso}\right)}\;g\;dV_{\left(\mathrm{LC}\right)}$$

(3) $$dF_{\mathrm{cf}}=-\rho_{\left(\mathrm{LC}\right)}\;\omega \times \left[\omega \times r_{\left(\mathrm{dV}\right)}\right]dV_{\left(\mathrm{LC}\right)}$$

(4) $$dF_{\mathrm{bcf}}=\rho_{\left(\mathrm{Iso}\right)}\omega \times \left[\omega \times r_{\left(\mathrm{dV}\right)}\right]dV_{\left(\mathrm{LC}\right)}$$

where *ρ_(LC)_* and *ρ_(Iso)_* represent the volumetric mass densities of the liquid crystalline tactoid and the isotropic phase, respectively. Generally, *ρ_(LC)_* is slightly higher than *ρ_(Iso)_* by 0.1–0.2% as cellulose nanocrystals (with a density of ≈1.5–1.6 g cm^−3^) are more densely packed inside tactoids than in isotropic regions due to their ordered alignments in liquid crystalline phases.^[^ ^43^ ^]^ When the centrifugal acceleration field **a**_cf_ = − **ω** × [**ω** × **r**] is significantly stronger than the gravitational acceleration field **g**, the movement of tactoids will be predominantly determined by the vector sum of the fictitious centrifugal force and the centrifugal buoyancy force (here the differences in **r**_(dV)_ have been overlooked):

(5) $$\Sigma F_{\mathrm{centrifugal}}=-\left[\rho_{\left(\mathrm{LC}\right)}-\rho_{\left(\mathrm{Iso}\right)}\right]\omega \times \left[\omega \times r\right]V_{\left(\mathrm{tactoid}\right)}$$

Therefore, tactoids will move radially outward from the rotation axis of the rotor, and eventually form a continuous liquid crystalline phase at the end of the centrifuge tube by coalescence (Figure 2G).

## 4 Speed Control of Redox‐Initiated Free‐Radical Polymerization Processes at Room Temperature

**Figure 3:**
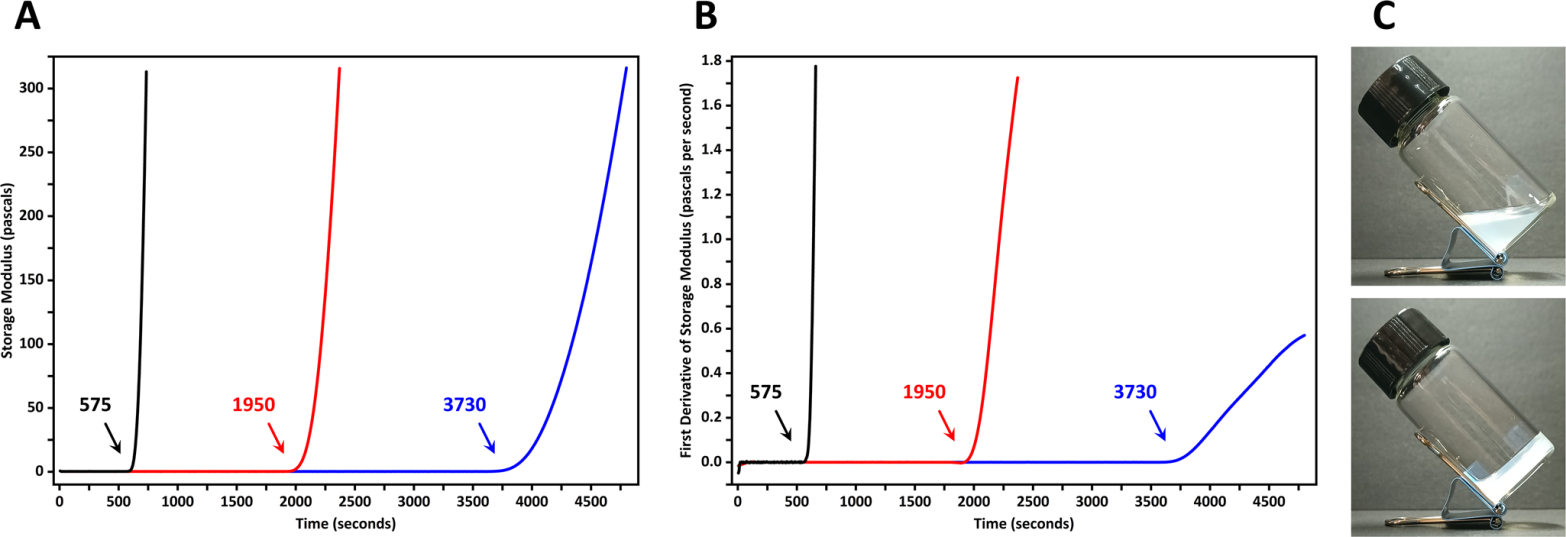
Kinetics of the polymerization of acrylamide initiated by the redox reactions between tert‐butyl hydroperoxide (30.96 mmol L^−1^) and thiourea (black: 19.71 mmol L^−1^; red: 10.51 mmol L^−1^; blue: 6.57 mmol L^−1^). A) The storage modulus G′ with respect to time. B) The first derivatives of the storage modulus with respect to time (dG'/dt). C) Photographs of an aqueous dispersion containing cellulose nanocrystals and precursors of polyacrylamide before (upper) and after (lower) polymerization. The external diameter of the glass vial was 22.09 mm.

The redox‐initiated free‐radical polymerization process used for in situ immobilization of self‐assembled microstructures was investigated by rheological measurements. In a typical experiment, a clear aqueous solution (acidified to pH 3.0 with hydrogen chloride and deoxygenated by bubbling nitrogen) of acrylamide (1.407 mol L^−1^), *N*,*N*'‐methylenebis(acrylamide) (64.86 mmol L^−1^), and thiourea (6.57 mmol L^−1^) was homogeneously mixed with tert‐butyl hydroperoxide (30.96 mmol L^−1^) and then immediately loaded into the gap (1.0 mm in thickness) between parallel lower stationary and upper rotating plates (20 mm in diameter) of a rotary rheometer. Rheological analysis was performed under a nitrogen atmosphere at a frequency of 1.0 Hz and a constant temperature of 298 K. The mixed solution showed a storage modulus (G') lower than 0.30 pascals within the first 3670 s; afterward, its storage modulus increased to 101.0 Pa at 4340 s and 302.0 Pa at 4775 s, indicating the formation of polyacrylamide chains that raised the viscosity of the solution (**Figure** **3**A; Figure S5A–C, Supporting Information). The first derivative of the storage modulus with respect to time (dG'/dt) remained lower than 0.008 pascals per second during the first 3725 s, which exceeded 0.01 Pa s^−1^ at 3730 s and kept rising after this time point (Figure 3B; Figure S5D–F, Supporting Information). For a mixture solution containing 10.51 mmol L^−1^ of thiourea, the storage modulus was below 0.30 Pa within the first 1935 s, then increased to 101.5 Pa at 2215 s and 307.3 Pa at 2365 s. The dG'/dt of this system was lower than 0.006 Pa s^−1^ during the first 1945 s; it exceeded 0.01 Pa s^−1^ at 1950 s, and thenceforth continuously rose higher. Moreover, for a polymer precursor solution containing 19.71 mmol L^−1^ of thiourea, the storage modulus remained below 0.30 Pa and the dG'/dt was lower than 0.005 Pa s^−1^ within the first 570 s, then dG'/dt exceeded 0.01 Pa s^−1^ at 575 s and kept increasing thereafter, while G' soared to 104.4 Pa at 685 s and 313.2 Pa at 735 s. These results indicated that the viscosity of the system stayed constantly (the first derivative of the storage modulus with respect to time was almost 0 Pa s^−1^) at a sufficiently low level for a period of time before the initiation of free‐radical polymerization, then it dramatically increased and rapidly transformed the originally liquid‐state dispersion into a nonflowing hydrogel. During this low‐viscosity period of time, the duration of which was controllable as it could be lengthened by decreasing the concentration of thiourea, self‐assembly of colloidal anisotropic particles and evolution of soft ordered microstructures were not hindered by long polymer chains. Afterward, once the polymerization process was initiated, the viscosity of the dispersion began to rise quickly due to the in situ formation of three‐dimensionally crosslinked polyacrylamide networks, which eventually kinetically terminated the evolution of liquid crystalline phases by preventing the movement and rotation of the rod‐shaped mesogenic nanoparticles.

Starting points of in situ free‐radical polymerization in aqueous CNC dispersions were also visually observable as the transparency of dispersion would suddenly decrease when its viscosity began to increase (Figure S5G–J, Supporting Information); several tens of seconds or a few minutes (depending of the concentrations of tert‐butyl hydroperoxide and thiourea) later, the system completely transformed from a translucent liquid into an opaque nonflowing hydrogel (Figure 3C). According to this visual criterion, effects of thiourea on the polymerization kinetics of acrylamide in CNC dispersions were evaluated. A series of aqueous suspensions containing cellulose nanocrystals (57.6 mg L^−1^), acrylamide (1.407 mol L), *N*,*N*'‐methylenebis(acrylamide) (64.86 mmol L^−1^), and various concentrations of thiourea (3.94 to 78.82 mmol L^−1^) were prepared; after deoxygenation by bubbling nitrogen for 60 s, tert‐butyl hydroperoxide (30.96 mmol L^−1^) was added, then the suspensions were left standing in sealed glass vials at 298 kelvins for 90 min. With 3.94, 6.57, 10.51, 13.14, 15.76, 19.71, 26.27, 39.41, 59.12, and 78.82 mmol L^−1^ of thiourea, initiation of polymerization was observed 83, 62, 32, 25, 20, 10, 8, 5, 3, and 2 min after the addition of tert‐butyl hydroperoxide, respectively, which matched well with the moments when viscosity of the systems began to dramatically increase (dG'/dt > 0.01 Pa s^−1^) in rheological tests (**Table** **1**).

**Table 1:** Initiation times of free‐radical polymerization of acrylamide initiated by redox reactions between tert‐butyl hydroperoxide and thiourea of different concentrations.

(CH_3_)_3_C─O─O─H [mmol L^−1^]	(H_2_N)_2_C═S [mmol L^−1^]	Transparency Decrease	Viscosity Increase [dG'/dt > 0.01 Pa s^−1^]
30.96	3.94	83 min	
30.96	6.57	62 min	3730 s (62 min 10 s)
30.96	10.51	32 min	1950 s (32 min 30 s)
30.96	13.14	25 min	
30.96	15.76	20 min	
30.96	19.71	10 min	575 s (9 min 35 s)
30.96	26.27	8 min	
30.96	39.41	5 min	
30.96	59.12	3 min	
30.96	78.82	2 min	

## 5 Effects of Centrifugal Acceleration Fields on the Structural Evolution of Liquid Crystalline Tactoidal Microphases in Low‐Viscosity Colloidal Dispersions

**Figure 4:**
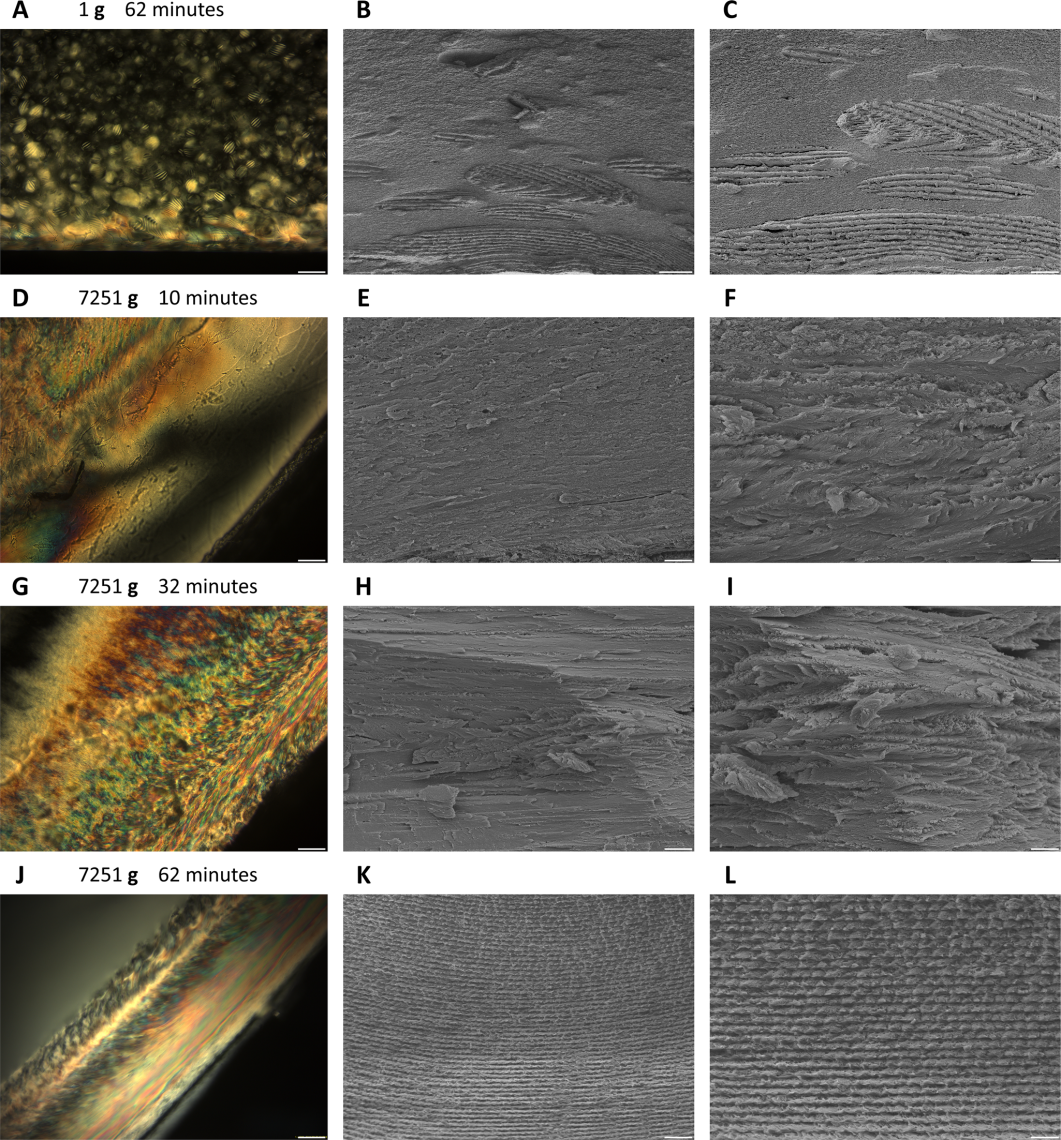
Polarized optical microscopy (A,D,G,J; with two linear polarizers oriented in the North‐South and East‐West directions, illuminated by transmitted white light) and cross‐sectional scanning electron microscopy (B,C,E,F,H,I,K,L) images showing liquid crystalline microstructures self‐assembled by cellulose nanocrystals after 10 min (D‐F), 32 min (G‐I), and 62 min (A‐C, J‐L) of evolution in the gravitational field of the Earth (**g** = 9.8 m s^−2^) (A‐C) or a centrifugal acceleration field of 71061.15 m s^−2^ (7251 **g**) (D‐L). Scale bars: (A,D,G,J) 100 µm, (B) 20 µm, (C) 10 µm, (E,H,K) 5 µm, (F,I,L) 2 µm.

With the assistance of the time‐controllable redox‐initiated in situ free radical polymerization, the emergence and transformations of self‐assembled liquid crystalline microstructures in centrifugal acceleration fields of different strengths could be terminated at predetermined evolution stages (e.g., after 10, 32, or 62 min) and immobilized within crosslinked polyacrylamide matrices for optical and electron microscopy examinations. When only affected by the gravity of the Earth, tactoids in a stationary aqueous dispersion of cellulose nanocrystals settled vertically downward. As revealed by polarized optical microscopy images of thin cross‐sections of cellulose nanocrystal dispersions (5.6 wt.%) polymerized 62 min after homogenization (denoted as 1 g_62 min), discrete chiral nematic tactoids with random dimensions, positions, and helical axis orientations were formed throughout the samples (**Figure** **4**A; Figure S6, Supporting Information). At this stage, tactoids started to gather together and aggregate near the bottom of the dispersions, where geometrically irregular anisotropic microregions with minimal thicknesses of ≈170–240 microns and defective structures began to appear. However, sedimentation and integration of tactoids had not completed since the average movement velocity of these ordered microdroplets was very slow in the 9.8 m s^−2^ gravitational acceleration field. Cross‐sectional scanning electron microscopy (SEM) images of dried CNC/polyacrylamide composite gels confirmed the existence of large amounts of free discrete tactoids, incompleteness of isotropic/anisotropic interfaces, and presence of topological defects in incipient liquid crystalline microphases (Figure 4B,C; Figure S7, Supporting Information). Similarly, for shorter durations of structural evolution, the aggregation and coalescence processes of tactoids were less advanced. In CNC dispersions immobilized after motionless standing for 10 and 32 min, only separate tactoids could be observed, while no large‐volume anisotropic regions were found at the bottom (Figures S8 and S9, Supporting Information).

In a centrifugal acceleration field of ≈7251 g (71061.15 m s^−2^, generated by an angular velocity of 942.48 rad s^−1^ in the vertical direction and a radius of 80 mm), the horizontally outward off‐axis movements of tactoids were significantly faster than the gravity‐driven sedimentation, therefore they were severely stretched, distorted, and even torn apart by shear forces from ordered/disordered phase interfaces. These tactoids and their broken pieces could not maintain the ellipsoidal geometry originally shaped by interfacial tension forces that tended to minimize interfacial areas of ordered microdomains. As revealed by polarized optical microscopy images of a cellulose nanocrystal dispersion immobilized 10 min after homogenization by redox‐initiated in situ free radical polymerization (denoted as 7251 g_10 min), liquid crystalline tactoids were deformed by shear forces and blended into the continuous isotropic phase to form a birefringent mixture (Figure 4D). Cross‐sections of freshly‐cracked dried gels were examined under scanning electron microscopy, where geometrically irregular microdomains showing highly‐defective chiral nematic superstructures, randomly oriented helical axes, and unclear boundary interfaces were observed (Figure 4E,F; Figure S10, Supporting Information). After 32 min of evolution, most tactoids had settled to the bottom of the dispersion and coalesced into a long‐range continuous liquid crystalline phase below (with respect to the direction of the local acceleration field) the isotropic phase (Figure 4G). At this stage, the interface between isotropic and anisotropic phases was still under development and appeared as an intermediate zone exhibiting gradually increased birefringence from the disordered side to the ordered side (i.e., away from the rotation axis). The anisotropic phase consisted of misaligned chiral nematic layers with randomly distributed orientations and helical pitches, among which numerous topological defects were formed (Figure 4H,I; Figure S11, Supporting Information). Complete differentiation between liquid crystalline tactoids and disordered regions was observed in cellulose nanocrystal dispersions immobilized after 62 min of evolution (denoted as 7251 g_62 min), where a clear and sharp interface perpendicular to the centrifugal acceleration fields (parallel to the rotation axis) had been thoroughly established to separate a strongly birefringent anisotropic phase closer to the liquid/solid interface (near the end of the centrifuge tube) from a non‐birefringent isotropic phase closer to the rotation axis (Figure 4J; Figure S12, Supporting Information). Under cross‐sectional scanning electron microscopy, the anisotropic phase was discovered to be composed of periodically repeated parallel chiral nematic layers with their helical axes unidirectionally aligned along the centrifugal acceleration fields, i.e., the pseudo‐nematic planes were parallel to the flat liquid–solid interface at the end of the centrifuge tube due to the tangential (planar) anchoring of rod‐shaped nanoparticles at the boundary (Figure 4K). At high magnifications, individual mesogens of cellulose nanocrystals were directly observable as rod‐shaped elongated nanoparticles, which rotated by 180 degrees (i.e., a half helical pitch) from one side to the other in each left‐handed chiral nematic layer (Figure 4L; Figure S13, Supporting Information). Possessing nearly identical plane orientations and helical pitches, the spatial organization of these lamellate chiral nematic bands was almost perfect, and only little amounts of topological defects could be found. This phenomenon suggested that structural transformations and thermodynamic relaxation processes in continuous liquid crystalline phases were not kinetically stopped or arrested even in the absence of discrete tactoids as all of these anisotropic microdroplets had already merged together. After completion of phase separation, rearrangements of chiral nematic layers and reorientation of mesogenic nanoparticles continued to progress to achieve minimization of the overall free energy of the system by healing topological defects showing higher free energy per unit volume due to symmetry breaking and frustrations of orderliness,^[^ ^44^, ^45^ ^]^ and theoretically a perfect chiral nematic superstructure would be eventually formed throughout the ordered phase.

Degrees of structural orderliness of liquid crystalline phases in dried 7251 g_10 min, 7251 g_32 min, and 7251 g_62 min composite hydrogels were qualitatively compared through 2D discrete Fourier transformations (2D‐DFT) of cross‐sectional scanning electron microscopy images. In the logarithmic magnitude spectrum of a 7251 g_62 min sample, a series of high‐intensity spots were periodically spaced on the vertical axis and symmetrically positioned relative to the center of the magnitude spectrum in the frequency domain. These bright spots represented vertically propagating sinusoidal waves of a specific frequency in the spatial domain, which indicated the existence of horizontally oriented structural characteristics that replicated periodically in the vertical direction in the real space.^[^ ^46^ ^]^ By using band‐pass filters in the frequency domain, higher‐frequency signals from small‐sized nanoparticles as well as lower‐frequency signals from highlights and shadows were removed, thus the arrangements of chiral nematic layers could be selectively extracted and then reconstructed in the spatial domain by 2D inverse discrete Fourier transformations (2D‐IDFT), where 7251 g_62 min composite gels showed significantly higher degrees of orderliness than the 7251 g_32 min and 7251 g_10 min samples (Figure S14, Supporting Information).

**Figure 5:**
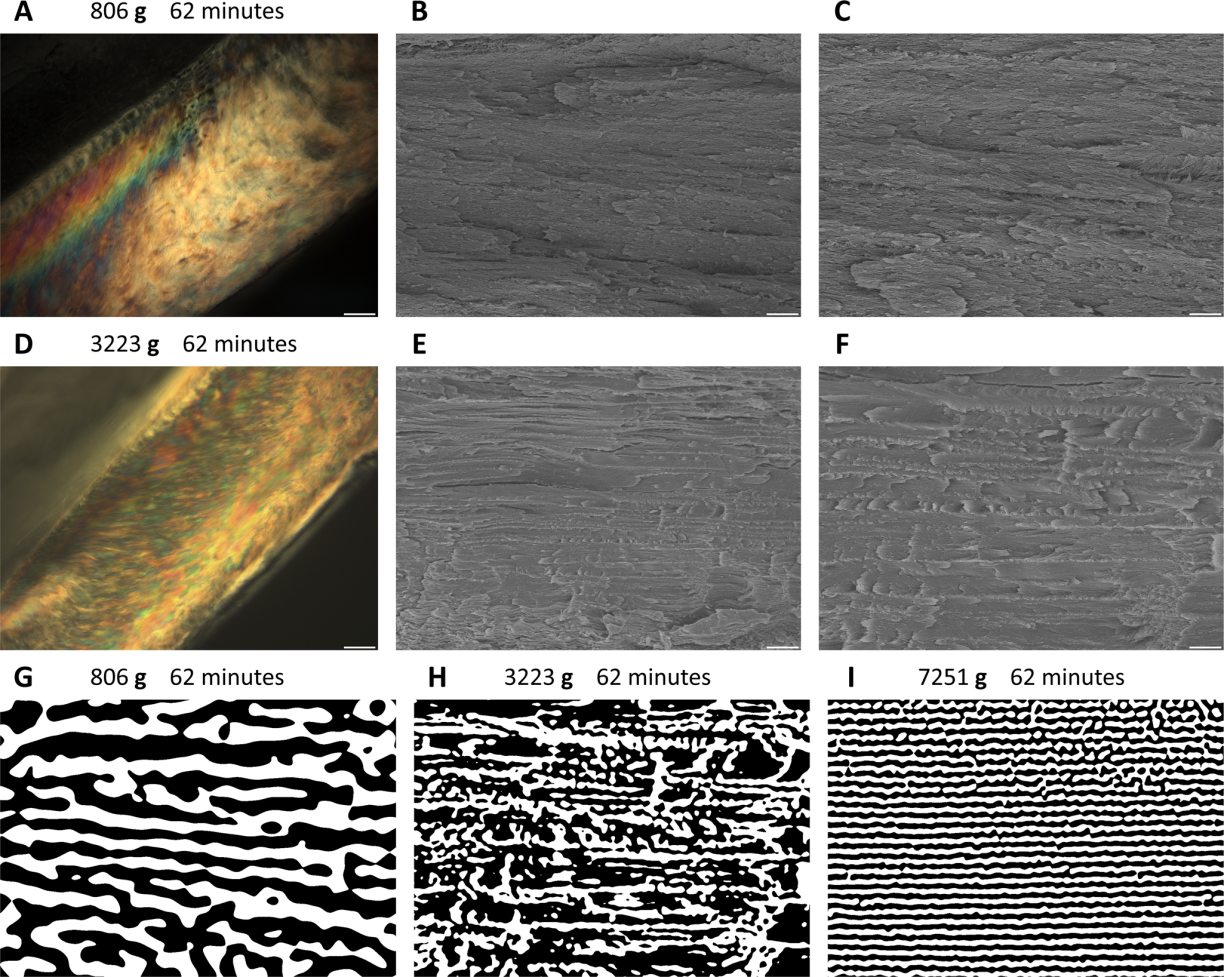
Polarized optical microscopy (A,D; with two linear polarizers oriented in the North‐South and East‐West directions, illuminated by transmitted white light) and cross‐sectional scanning electron microscopy (B,C,E,F) images showing liquid crystalline microstructures self‐assembled by cellulose nanocrystals after 62 min of evolution in centrifugal acceleration fields of 7895.68 m s^−2^ (≈806 g) (A‐C) and 31582.73 m s^−2^ (≈3223 g) (D‐F). Arrangements of chiral nematic layers in the ordered phases of 806 g_62 min (G), 3223 g_62 min (H), and 7251 g_62 min (I) samples were extracted using band‐pass filters in the frequency domain after 2D discrete Fourier transforms, then reconstructed in the spatial domain by inverse discrete Fourier transforms. Scale bars: (A,D) 100 µm, (B,E) 5 µm, (C,F) 2 µm.

Influences of the strengths of acceleration fields on self‐assembly kinetics were also experimentally investigated. While fixing the evolution time at 62 min, liquid crystalline structures formed by cellulose nanocrystals in centrifugal acceleration fields of 31582.73 m s^−2^ (≈3223 g, 628.32 rad s^−1^) and 7895.68 m s^−2^ (≈806 g, 314.16 rad s^−1^) were captured in crosslinked polyacrylamide matrixes by in situ redox‐initiated free radical polymerization (named as 3223 g_62 min and 806 g_62 min, respectively). Phase separation was completed in both samples as confirmed by polarized optical microscopy (**Figure** **5**A,D). Under cross‐sectional scanning electron microscopy, liquid crystalline phases in 3223 g_62 min samples showed significantly higher degrees of ordering than those in dried 806 g_62 min hydrogels (Figure 5B,C,E,F; Figures S15 and S16, Supporting Information), but neither of them was more ordered than the unidirectionally aligned and periodically repeated chiral nematic layers in 7251 g_62 min samples. Differences in the degrees of structural orderliness caused by the strengths of centrifugal acceleration fields were further analyzed by 2D‐DFT and 2D‐IDFT with the assistance of band‐pass frequency filters (Figure 5G–I; Figure S17, Supporting Information).

## 6 Increased Transformation Kinetics and Structural Orderliness: High‐Viscosity Colloidal Liquid Crystals in Strong Centrifugal Acceleration Fields

**Figure 6:**
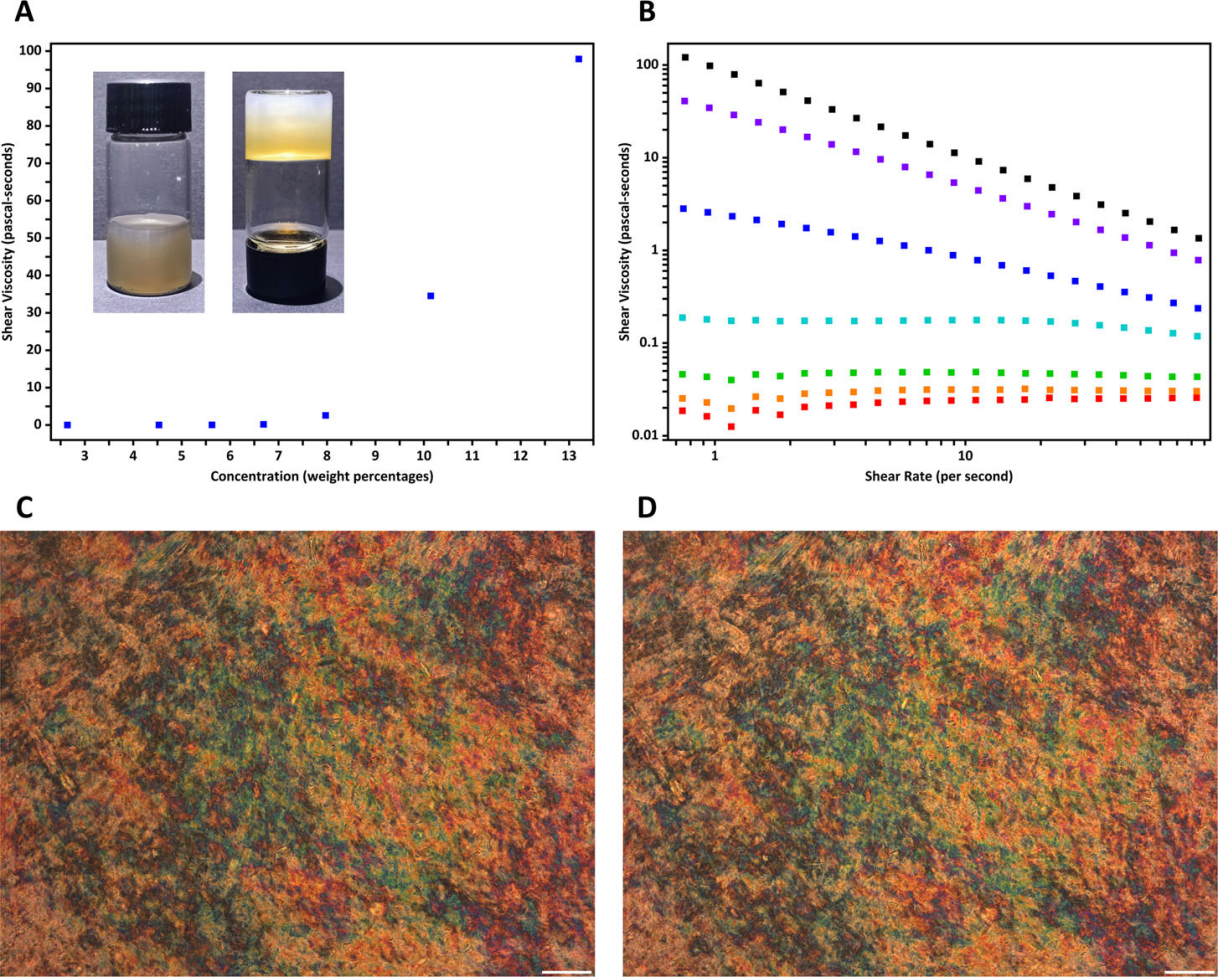
A) The shear viscosity of colloidal aqueous dispersions of cellulose nanocrystals with different concentrations. Photographs of a 10.142 wt.% dispersion are shown as insets (the external diameter of the glass vial was 18.33 millimeters). B) The shear viscosity of 2.640 wt.% (red), 4.531 wt.% (orange), 5.625 wt.% (green), 6.690 wt.% (cyan), 7.971 wt.% (blue), 10.142 wt.% (violet), and 13.200 wt.% (black) cellulose nanocrystal dispersions measured in relation to the shear rate. C,D) Polarized optical microscopy images showing the existence of topological defects in the same region of a 10.142 wt.% aqueous dispersion of cellulose nanocrystals before and after stationary standing for 48 h. The sample was placed between two linear polarizers oriented in the North‐South and East‐West directions. Scale bars: (C,D) 100 µm.

It has long been known that structural transformations in highly concentrated (e.g., beyond 10% by weight) aqueous dispersions of cellulose nanocrystals (and many other lyotropic liquid crystals) are kinetically arrested due to their high viscosity, which turns the colloidal dispersions into glassy or gelated states and significantly hinders the translational and rotational movements of mesogenic nanoparticles or molecules.^[^ ^47^, ^48^, ^49^ ^]^ In our experiments, the shear viscosity (at a shear rate of 1 per second) of cellulose nanocrystal dispersions with concentrations of 2.640%, 4.531%, 5.625%, 6.690%, 7.971%, 10.142%, and 13.200% (by weight) was measured to be 0.01, 0.02, 0.04, 0.18, 2.57, 34.53, and 97.89 pa‐s (**Figure** **6**A,B; Figure S18, Supporting Information). Microstructures in highly viscous liquid crystals (> 10 wt.%) remained almost unchanged after prolonged motionless standing for 48 h (Figure 6C,D), and topological defects would hardly be healed once they have been formed.^[^ ^50^, ^51^ ^]^

**Figure 7:**
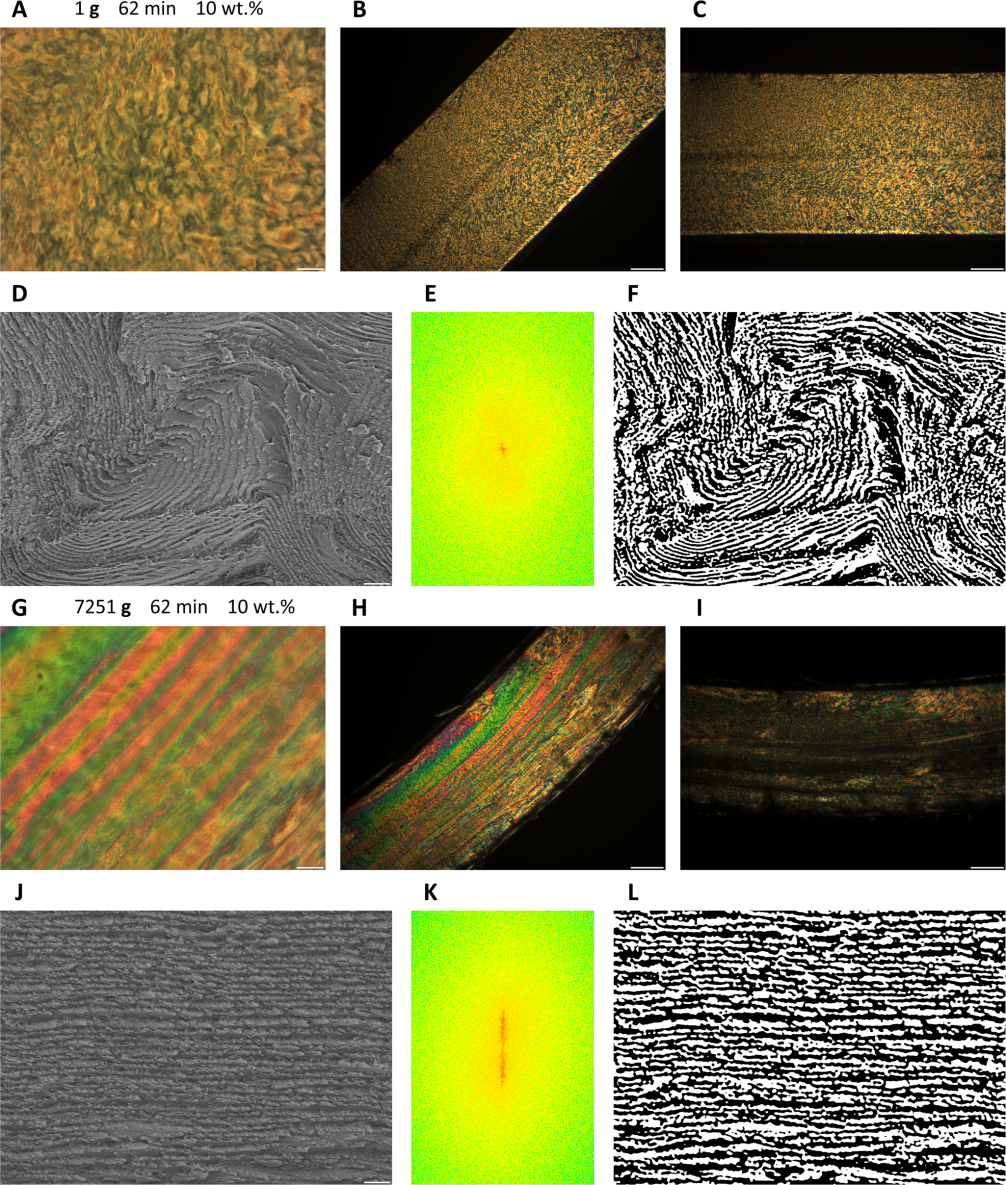
Cross‐sectional polarized optical microscopy (A‐C, G‐I; the samples were placed between two linear polarizers oriented in the North‐South and East‐West directions) and scanning electron microscopy (D,J) images of high‐concentration (≈10% by weight) aqueous dispersions of cellulose nanocrystals immobilized after 62 min of evolution in the gravitational field of the Earth (**g** = 9.8 m s^−2^) (A‐D) or a centrifugal acceleration field of 71061.15 m s^−2^ (7251 **g**) (G‐J). Logarithmic magnitude spectra (central regions; pseudo‐colored with 16 colors) of 1 g_62 min_10 wt.% (E) and 7251 g_62 min_10 wt.% (K) samples were calculated by 2D discrete Fourier transforms, then the arrangements of chiral nematic layers were extracted with band‐pass filters and reconstructed by inverse discrete Fourier transforms (F,L). Scale bars: (A,G) 50 µm, (B,C,H,I) 300 µm, (D) 5 µm, (J) 1 µm.

When examined between two perpendicularly oriented linear polarizers using optical microscopy, 10.142 wt.% cellulose nanocrystal dispersions captured by in situ polymerization after stationary standing for 62 min (denoted as 1 g_62 min_10 wt.%) in the gravitational acceleration field of the Earth (**g**) appeared as ensembles of numerous anisotropic microregions exhibiting irregular geometry, 10–100 µm dimensions, and nematic‐like birefringent textures with chaotically tangled director fields (**Figure** **7**A). No remarkable changes in birefringence were observed when the samples were rotated by 45 degrees between crossed polarizers, indicating that director field orientations of the anisotropic microregions were randomly distributed (Figure 7B,C; Figure S19, Supporting Information). Under scanning electron microscopy, these nematic‐like optical textures were discovered to be severely distorted chiral nematic layers (Figure 7D–F), among which large amounts of topological defects such as disclinations, dislocations, and grain‐boundary edges could be found (Figure S20, Supporting Information).

Contrarily, 10 wt.% aqueous dispersions of cellulose nanocrystals immobilized within crosslinked polyacrylamide networks by in situ redox‐initiated free radical polymerization after 62 min of evolution in centrifugal acceleration fields of 7251 **g** (denoted as 7251 g_62 min_10 wt.%) showed dramatically improved structural orderliness (Figure 7G). The centrifuged dispersions were still almost completely anisotropic (i.e., with nearly 100% volume fractions of anisotropic phases), while they consisted of long‐range monochromatically birefringent stripes with cross‐sectional lengths of up to 5 millimeters and thicknesses of up to 0.2 millimeters. Moreover, these anisotropic stripes were orderly aligned parallel to the gas–liquid and liquid–solid interfaces, or in other words, perpendicular to the centrifugal acceleration field vectors. When rotated between two crossed linear polarizers oriented at 0 and 90 degrees, the birefringent stripes exhibited maximum brightness at 45 and 135 degrees as well as minimum brightness at 0 and 90 degrees (Figure 7H,I; Figure S21, Supporting Information), indicating that the mesogenic nanorods were all arranged either parallel or perpendicular to the boundary interfaces. Cross‐sectional scanning electron microscopy images revealed that the liquid crystalline phases were tightly packed chiral nematic layers, which were unidirectionally aligned with the helical axis oriented parallel to the centrifugal acceleration fields (Figure 7J–L). Only small amounts of topological defects were observed within this highly ordered long‐range hierarchical superstructure, and average repetition periods (half helical pitches) of the chiral nematic layers seemed to have been remarkably compressed from ≈1200 to 250 nanometers by the strong acceleration fields (Figure S22, Supporting Information). Based on 2D discrete Fourier transform algorithms, logarithmic magnitude spectra for cross‐sectional SEM images of dried 1 g_62 min_10 wt.% and 7251 g_62 min_10 wt.% composite hydrogels were calculated (Figure 7E,K; enhanced with pseudo colors). The arrangements of chiral nematic layers were selectively extracted by band‐pass filters in the frequency domain, which was then reconstructed in the spatial domain through 2D inverse discrete Fourier transformations (Figure 7F,L; Figure S23, Supporting Information). Within a limited time of 62 min, the strong centrifugal acceleration fields of 7251 **g** significantly decreased the volumetric densities of topological defects such as disclinations, dislocations, and grain‐boundary edges, suggesting that the structural transition kinetics of liquid crystalline phases and relaxation (e.g., translational, vibrational, and rotational motions) rates of mesogenic colloidal nanoparticles in the high‐viscosity gelated dispersions were greatly accelerated, therefore the so‐called kinetically arrested states were overcome, and these glassy colloidal lyotropic liquid crystals behaved like thermotropic liquid crystalline species consisting of small molecules, which can spontaneously get topological defects healed to achieve minimization of the total free energy due to the much faster kinetic relaxation rates of the molecular mesogens.

**Figure 8:**
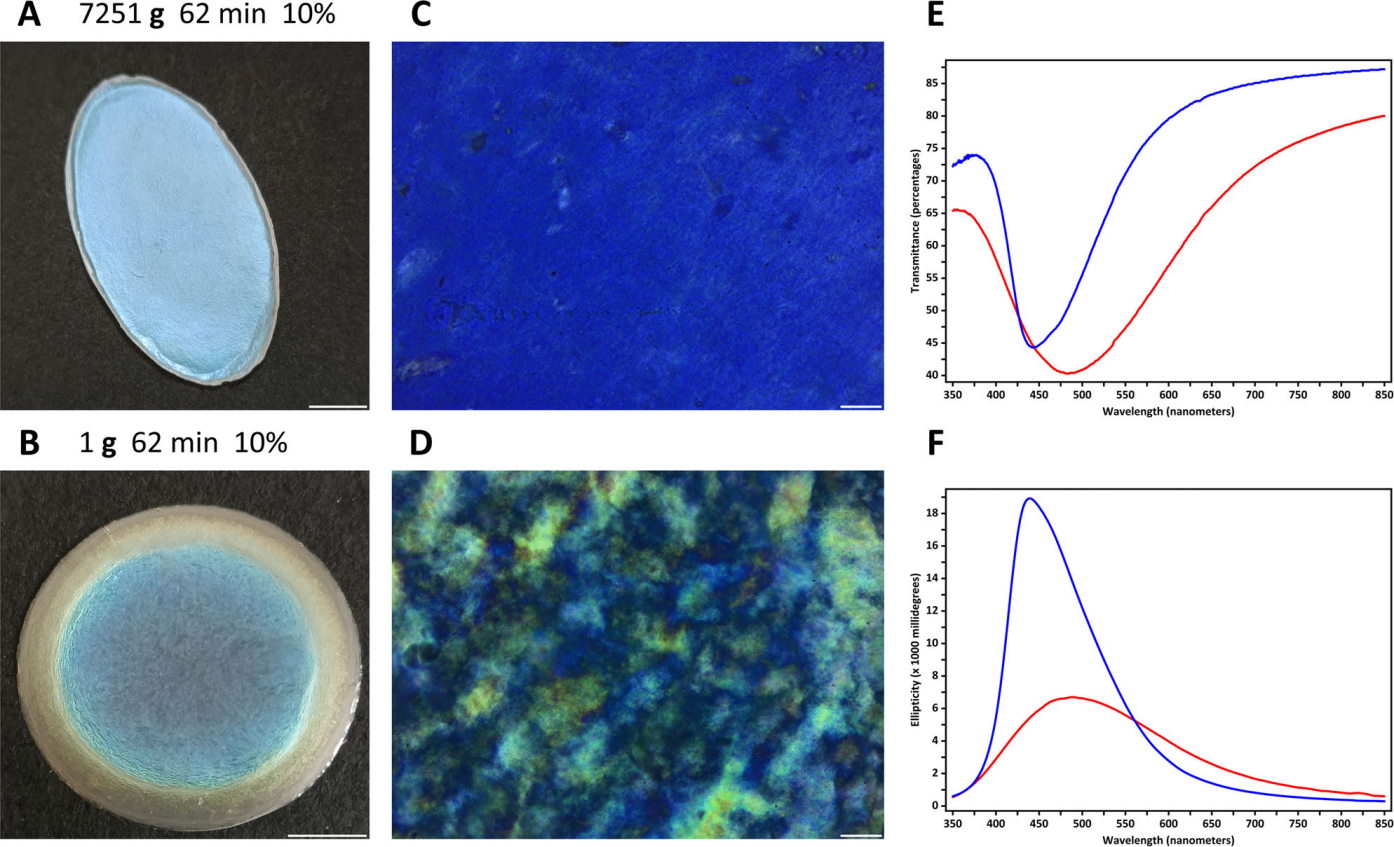
A–D) Photographs (A,B) and polarized optical microscopy images (C,D) of 7251 g_62 min_10 wt.% and 1 g_62 min_10 wt.% films. E,F) UV–vis transmission and circular dichroism spectra of 7251 g_62 min_10 wt.% (blue‐colored) and 1 g_62 min_10 wt.% (red‐colored) films. In (C) and (D), the samples were placed between two linear polarizers oriented in the North‐South and East‐West directions and illuminated by transmitted white light. Scale bars: (A,B) 5 mm, (C,D) 100 µm.

By lowering the concentrations of monomers and cross‐linkers (acrylamide 352 mmol L^−1^, *N*,*N*'‐methylenebis(acrylamide) 16.23 mmol L^−1^), the cellulose‐polyacrylamide composite hydrogels could be dried into solid‐state films with reduced thicknesses. Thin films obtained by drying 7251 g_62 min_10 wt.% hydrogels showed remarkably blue‐shifted iridescent colors (**Figure** **8**A,B) and more homogeneous liquid crystalline microstructures than those dried from 1 g_62 min_10 wt.% hydrogels (Figure 8C,D; Figure S24, Supporting Information). The decreases in chiral nematic helical pitches and improvements in structural uniformity resulting from strong centrifugal acceleration fields were also confirmed by ultraviolet‐visible extinction and circular dichroism spectroscopy, where the 7251 g_62 min_10 wt.% films exhibited narrower resonance reflection bands and significantly stronger ellipticity signals than 1 g_62 min_10 wt.% samples (Figure 8E,F).

## 7 Conclusion

In summary, the effects of strong centrifugal acceleration fields up to 7251 **g** (71061.15 m s^−2^) on the microscopic kinetics of entropy‐driven self‐assembly of rod‐shaped anisotropic nanoparticles in aqueous colloidal dispersions were experimentally investigated. With the assistance of a time‐controlled room‐temperature redox‐initiation system using non‐ionic water‐soluble oxidants and reductants of tert‐butyl hydroperoxide and thiourea, transformations of soft ordered microstructures were kinetically terminated at different evolution stages after predetermined periods of time by in situ free‐radical polymerization at 298 kelvins, and the self‐assembled anisotropic phases of mesogenic nanorods were immobilized within cross‐linked polyacrylamide matrixes. According to cross‐sectional optical and scanning electron microscopy observations, the rates of many structural transition processes in colloidal lyotropic liquid crystals were greatly dependent on the strengths of external acceleration fields, such as the movements and coalescence of liquid crystalline tactoids, the formation of long‐range continuous smooth interfaces between ordered and disordered phases, the rearrangements of director field vectors toward minimization of the total free energy, and the spontaneous elimination of topological defects during relaxation. Thermodynamic equilibrium could be achieved after a short evolution time in lyotropic liquid crystals when driven by centrifugal acceleration fields that were thousands of times stronger than the gravity of the Earth (**g** = 9.8 m s^−2^). In acceleration fields of 71061.15 m s^−2^, macroscopic helical superstructures of periodically spaced parallel chiral nematic layers were quickly formed in high‐viscosity concentrated (10 wt.%) dispersions of cellulose nanocrystals, where the so‐called kinetically arrested glassy (or gelated) states were readily overcome, and the resultant liquid crystalline phases showed significantly improved structural orderliness compared with those obtained in the gravitational acceleration field of the Earth. Strong centrifugal acceleration fields may also lead to faster relaxation rates and higher structural orderliness in the large‐scale fabrication of hierarchically structured materials from lyotropic liquid crystals or other self‐assembling systems, where a practical problem would be how to remove solvents from liquid crystalline dispersions during high‐speed centrifugation while keeping the rotating rotor balanced. The phenomena observed in this study provided additional insights into the microkinetics and evolution mechanisms behind the self‐assembly behaviors of anisotropic colloidal nanoparticles and also suggested a simple method to dynamically control the microstructures of lyotropic liquid crystals as well as the derived functional nanomaterials. Moreover, according to the principle of equivalence in general relativity developed by Albert Einstein,^[^ ^52^ ^]^ the effects of a uniform acceleration field are indistinguishable from those of a homogeneous gravitational field, therefore the results of this study might also help to predict the self‐assembly behaviors of colloidal liquid crystals or other ordered soft matter near massive or compact astrophysical objects such as white dwarfs or neutron stars.

## 8 Calculations

For a rectangular image g(x,y) consisting of M rows and N columns, the (forward) 2D discrete Fourier transformation was conducted based on the following algorithm:^[^ ^53^ ^]^

(A1) F(u,v)=SUM{g(x,y)exp[−i2π(ux/M+vy/N)]}

where x = 0, 1, 2, …, M‐1; y = 0, 1, 2, …, N‐1; SUM means double summation; and i = (‐1)^(0.5).

The corresponding inverse discrete Fourier transformation algorithm was:

(A2) g(x,y)=(1/MN)SUM{F(u,v)exp[i2π(ux/M+vy/N)]}

where u = 0, 1, 2, …, M‐1 and v = 0, 1, 2, …, N‐1.

## Conflict of Interest

The authors declare no conflict of interest.

## Supporting information



Supporting Information

## Data Availability

The data that support the findings of this study are available from the corresponding author upon reasonable request.
