# Supplementary material for: Liquid Crystalline Self‐Assembly with Accelerated Kinetics and Higher Structural Orderliness in Centrifugal Acceleration Fields Beyond 7251 Times Gravity of Earth
Source: Adv Sci (Weinh). 2025 Apr 25;12(21):2415955. doi: 10.1002/advs.202415955 (PMC12140379; doi:10.1002/advs.202415955)
Supplement: Supplementary file 1 — Supporting Information [file ADVS-12-2415955-s001.pdf]

ADVANCED SCIENCE
Open Access

Supporting Information

for *Adv. Sci.*, DOI 10.1002/advs.202415955

Liquid Crystalline Self-Assembly with Accelerated Kinetics and Higher Structural Orderliness in Centrifugal Acceleration Fields Beyond 7251 Times Gravity of Earth

*Lingyan Xu, Hongbo Zhao and Pei-Xi Wang**

ELECTRONIC SUPPLEMENTARY INFORMATION FOR

Liquid Crystalline Self-Assembly with Accelerated Kinetics and Higher Structural Orderliness in Centrifugal Acceleration Fields Beyond 7251 Times Gravity of Earth

Lingyan Xu, Hongbo Zhao, Pei-Xi Wang*

L. Xu, H. Zhao, P.-X. Wang

School of Nano-Tech and Nano-Bionics

University of Science and Technology of China

96 Jinzhai Road, Hefei, Anhui, 230026, P. R. China

L. Xu, H. Zhao, P.-X. Wang

i-Lab

Suzhou Institute of Nano-Tech and Nano-Bionics of the Chinese Academy of Sciences

398 Ruoshui Road, Suzhou, Jiangsu, 215123, P. R. China

* E-mail: pxwang2020@sinano.ac.cn

EXPERIMENTAL METHODS

Materials

Acrylamide (98%, Sigma-Aldrich), N,N′-Methylenebis(acrylamide) (99%, Sigma-Aldrich), thiourea (99%, Sigma-Aldrich), tert-butyl hydroperoxide solution (70% by weight in water, Sigma-Aldrich), and sulfuric acid (95-98%, Sigma-Aldrich) were used as received.

Equipment

Transmission electron microscopy was performed on a Thermo Scientific Talos F200X Scanning/Transmission Electron Microscope. Polarized optical microscopy images were obtained from an NP620 Polarized Optical Microscope. Field emission scanning electron microscopy was performed on a Hitachi Regulus 8230 Ultra-high Resolution Scanning Electron Microscope. Rheological analysis was conducted on a Kinexus Pro+ Rheometer, where the storage modulus (G′) and loss modulus (G′′) values were measures every 5 seconds. Circular dichroism spectroscopy was performed on an Applied Photophysics Chirascan-plus circular dichroism spectrometer. Ultraviolet-visible transmission spectroscopy was conducted on a PerkinElmer LAMBDA 25 UV/Vis Spectrophotometer. Centrifugation experiments were carried out using a Benchtop High Speed Refrigerated Centrifuge TGL-16M.

A B

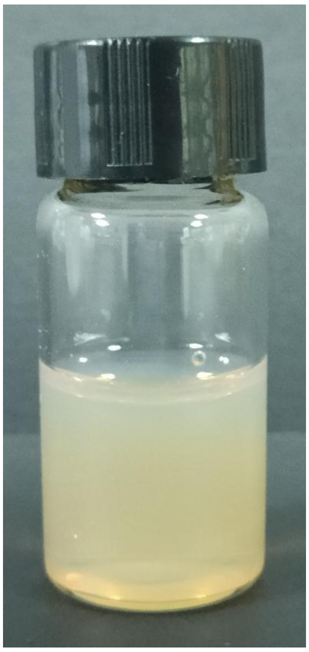

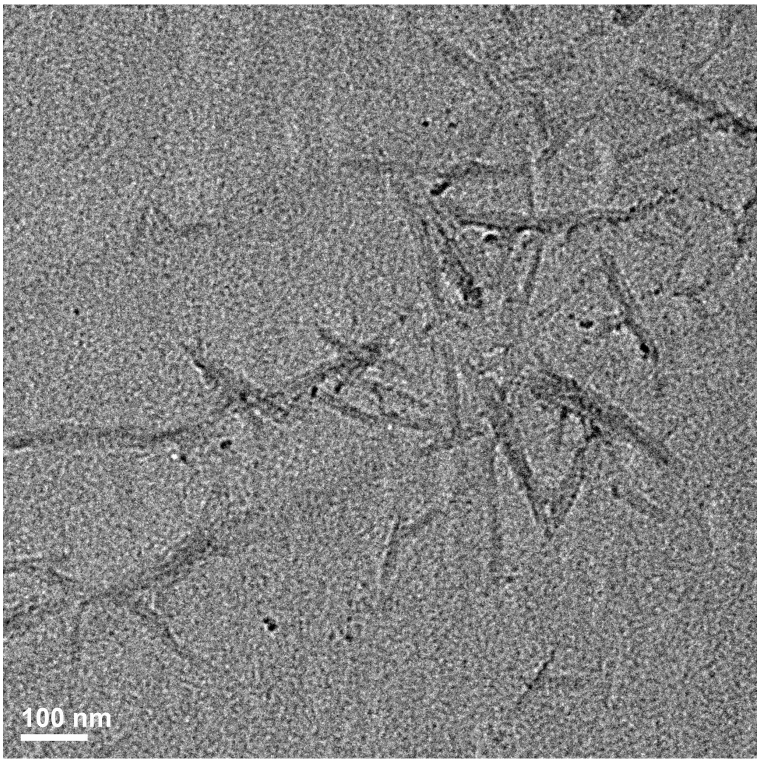

Figure S1. (**A**) A photograph showing an aqueous colloidal dispersion of cellulose nanocrystals (about 5% by weight). The external diameter of the glass vial was 18.33 millimeters. (**B**) A representative transmission electron microscopy image of cellulose nanocrystals.

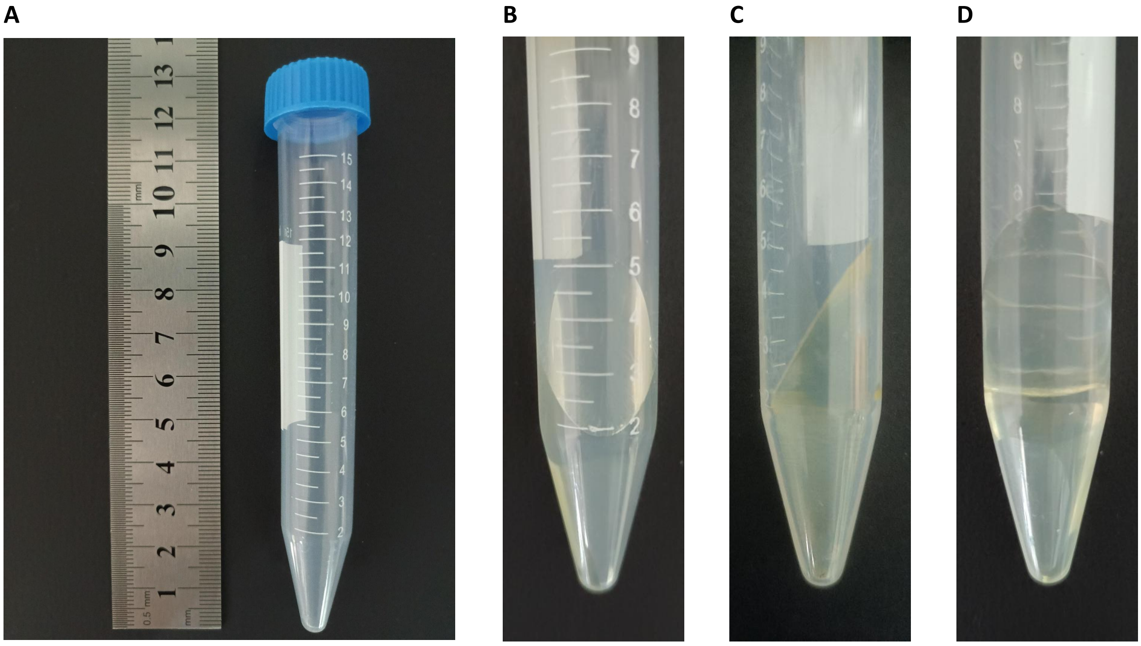

Figure S2. (**A**) A photograph of an empty centrifuge tube. (**B**-**D**) Photographs showing the front view (**B**), side view (**C**), and back view (**D**) of a centrifuge tube with a plane surface (formed by liquid epoxy resins that were solidified at the end of the tube during centrifugation at 9000 revolutions per minute) parallel to the rotation axis of the fixed-angle rotor.

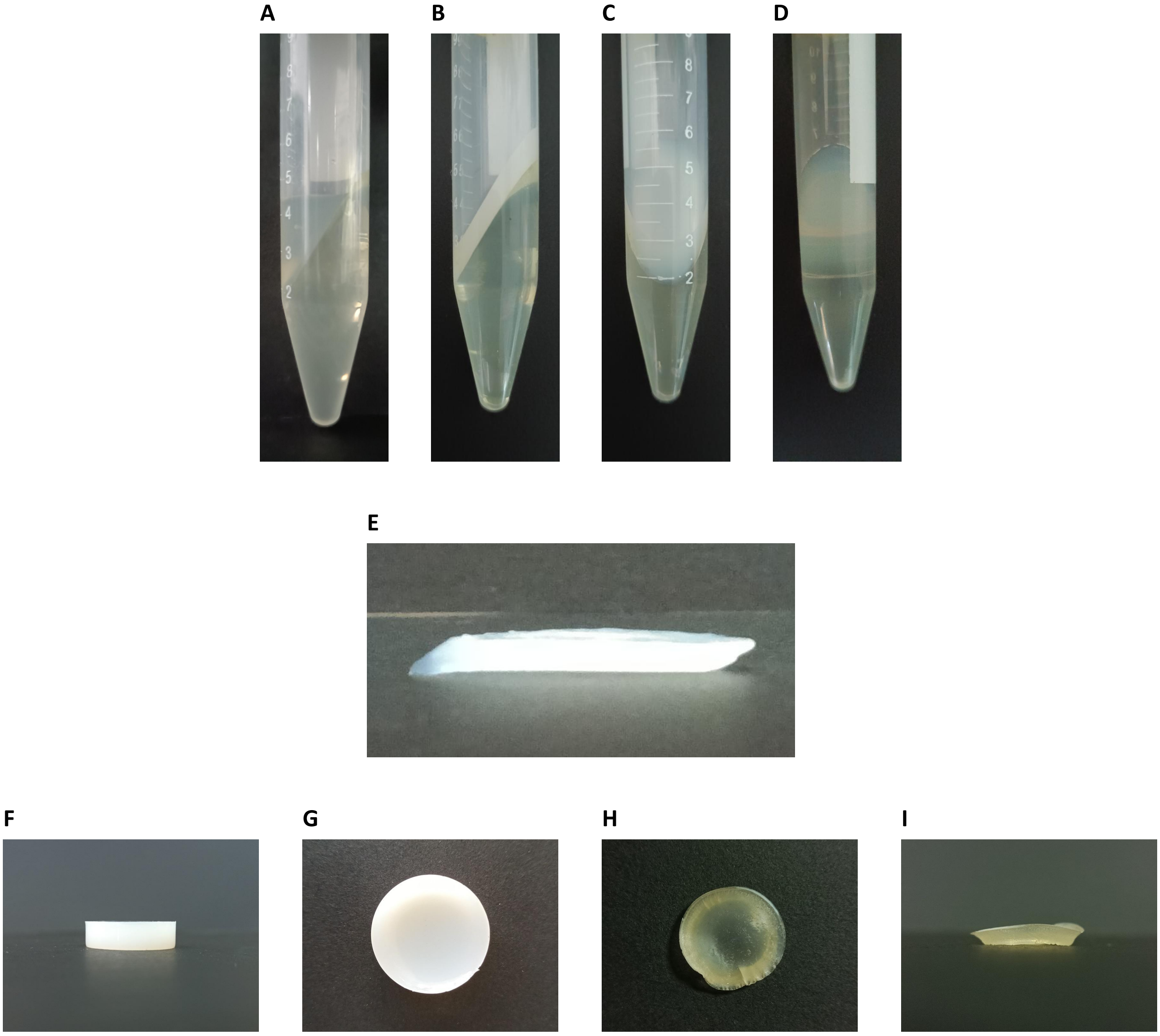

Figure S3. In a typical experiment, a homogeneous aqueous dispersion containing cellulose nanocrystals, acrylamide, N,N’-methylenebis(acrylamide), thiourea, and tert-butyl hydroperoxide was added into a centrifuge tube with a plane solid surface parallel to the rotation axis of the fixed-angle rotor (**A**). The tube was then placed into a centrifuge and the dispersion was centrifuged. Redox-initiated in-situ free-radical polymerization occurred during centrifugation at room temperature (about 298 kelvins), which transformed the aqueous dispersion into a crosslinked polyacrylamide hydrogel (**B**-**D**). The hydrogel could be taken out of the centrifuge tube while maintaining its integrity (**E**). (**F**-**I**) To evaluate the shrinkage of hydrogels during drying, a cylindrical-shaped hydrogel was prepared from the same aqueous dispersion without centrifugation, which showed a diameter of 19.24 mm and a thickness of 5.27 mm (**F**,**G**). After drying, its diameter and thickness decreased to 14.37 mm and 2.35 mm, respectively (**H**,**I**).

A

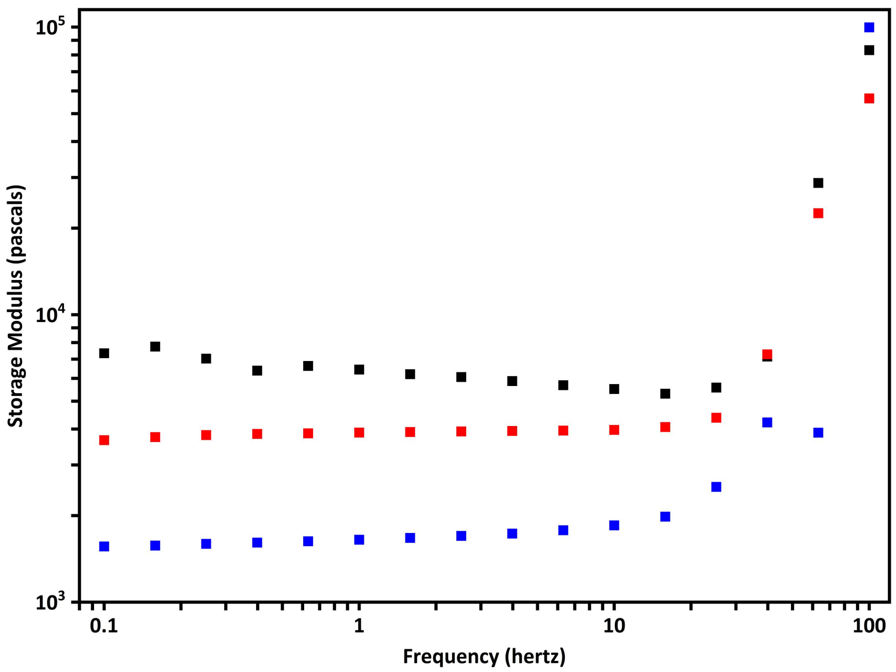

B

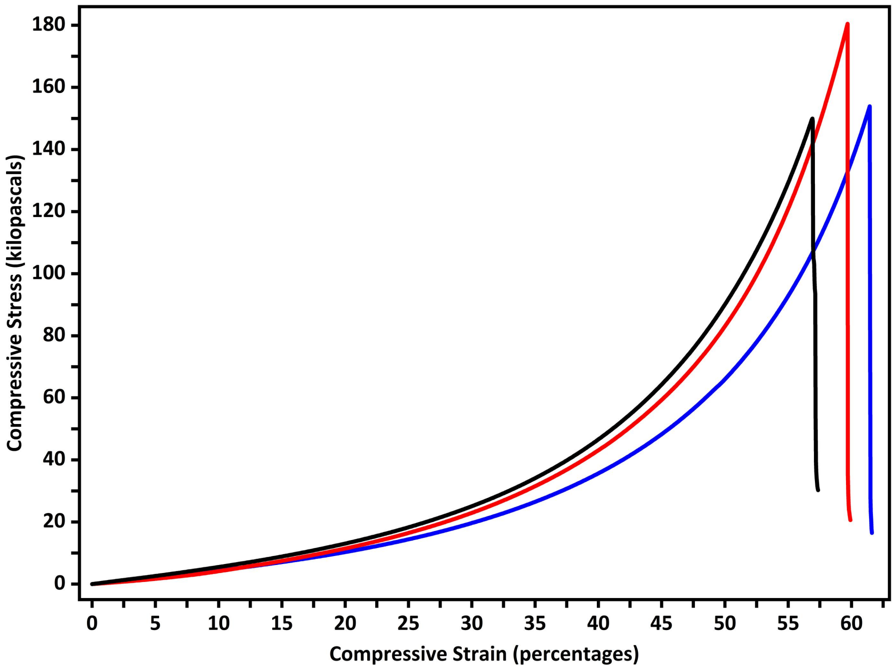

Figure S4. (**A**) Storage modulus values of polyacrylamide hydrogels with respect to frequency. The hydrogels were polymerized 10 minutes (black-colored curves), 32 minutes (red-colored curves), and 62 minutes (blue curves) after the addition of tert-butyl hydroperoxide. (**B**) Compressive stress - strain curves of these polyacrylamide hydrogels.

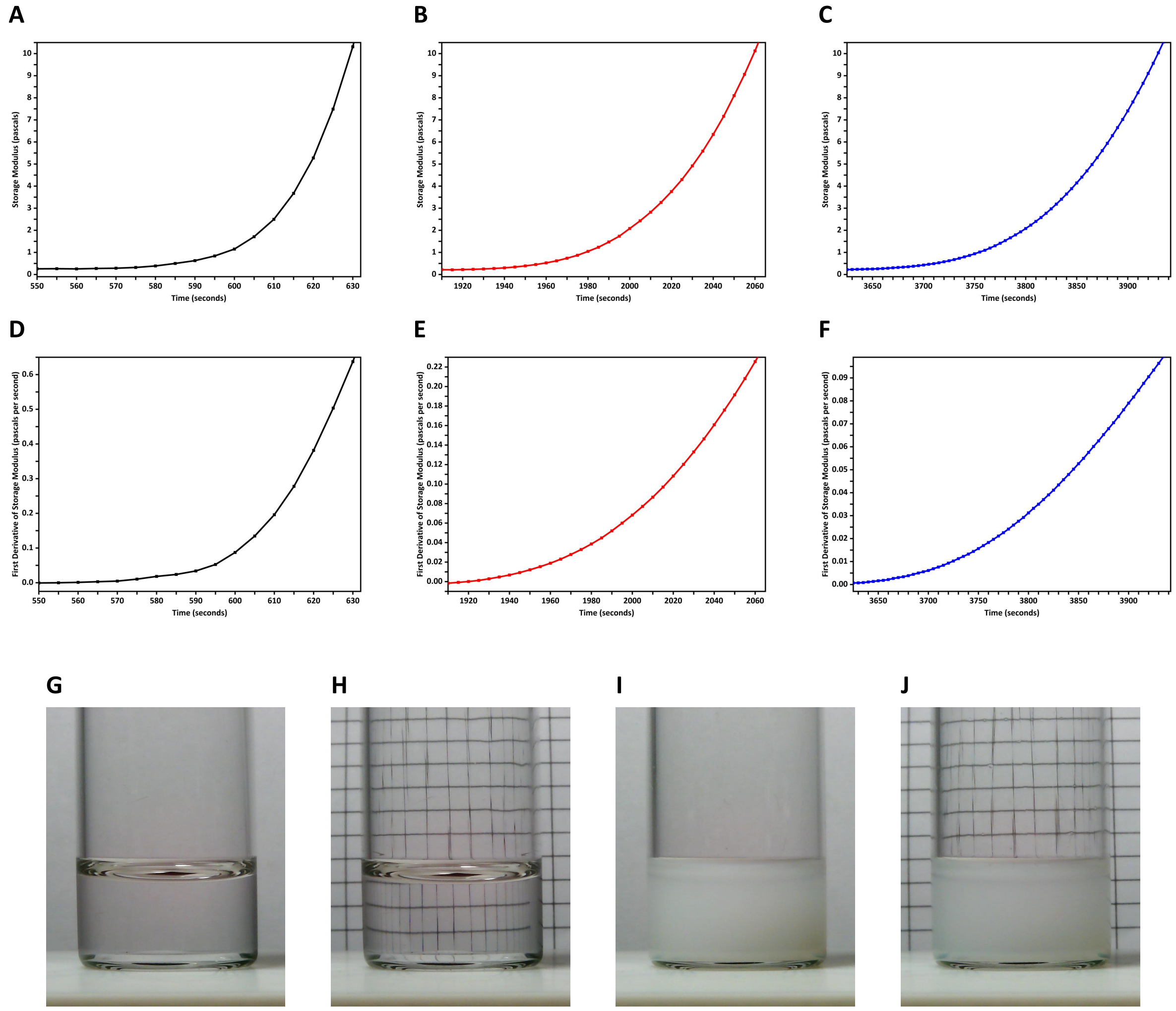

Figure S5. (**A**-**F**) Kinetics of the free-radical polymerization of acrylamide initiated by the oxidation-reduction reactions between tert-butyl hydroperoxide (30.96 mmol/L) and thiourea (black: 19.71 mmol/L; red: 10.51 mmol/L; blue: 6.57 mmol/L). (**A**-**C**) The storage modulus G' with respect to time. (**D**-**F**) The first derivatives of the storage modulus with respect to time (dG'/dt). These are the magnified views of the curves near the initiation times. (**G**-**J**) Photographs showing an aqueous solution containing acrylamide, N,N'-methylenebis(acrylamide), thiourea, and tert-butyl hydroperoxide before (**G**,**H**) and after (**I**,**J**) polymerization. In (**H**) and (**J**), a piece of white paper with black-colored grid lines was placed behind the glass vial. The external diameter of the glass vial was 22.32 millimeters.

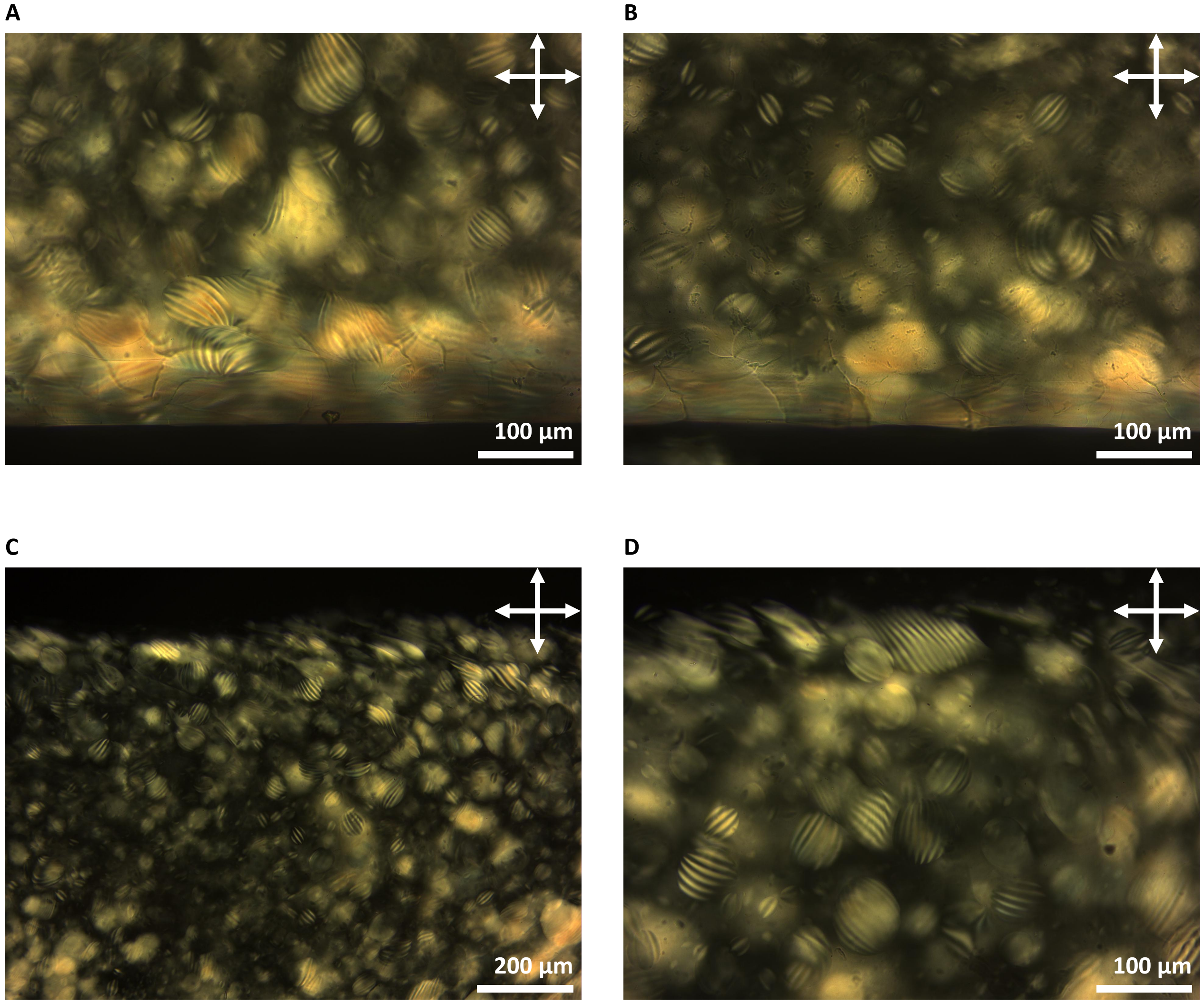

Figure S6. Polarized optical microscopy images (with two linear polarizers oriented in the North-South and East-West directions, illuminated by transmitted white light) showing chiral nematic liquid crystalline tactoids immobilized within the polyacrylamide matrix of a 1g_62minutes sample. The hydrogel sample was sliced with a razor blade to give fresh thin cross sections for cross-sectional microscopy observations.

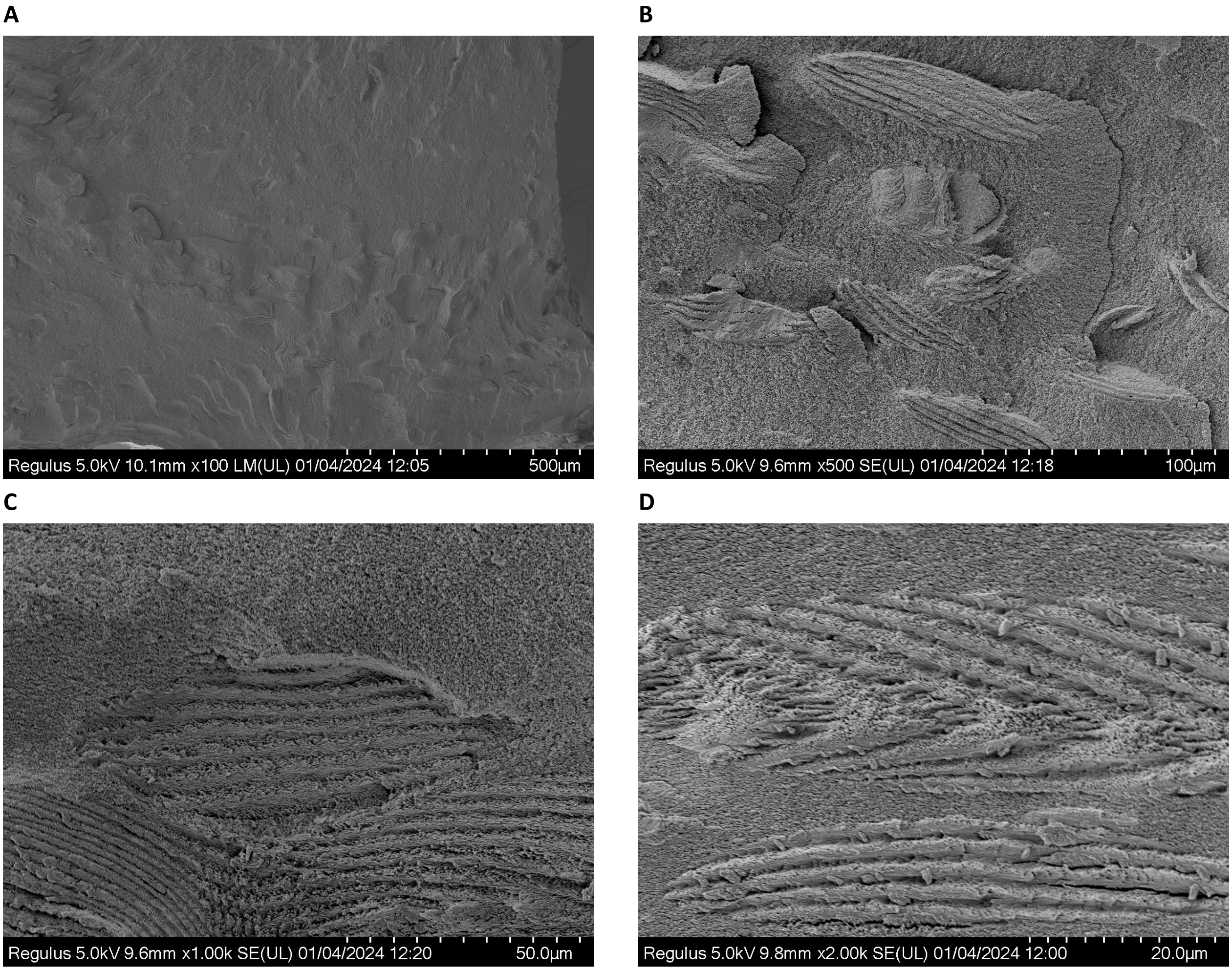

Figure S7. Cross-sectional scanning electron microscopy images of 1g_62minutes samples.

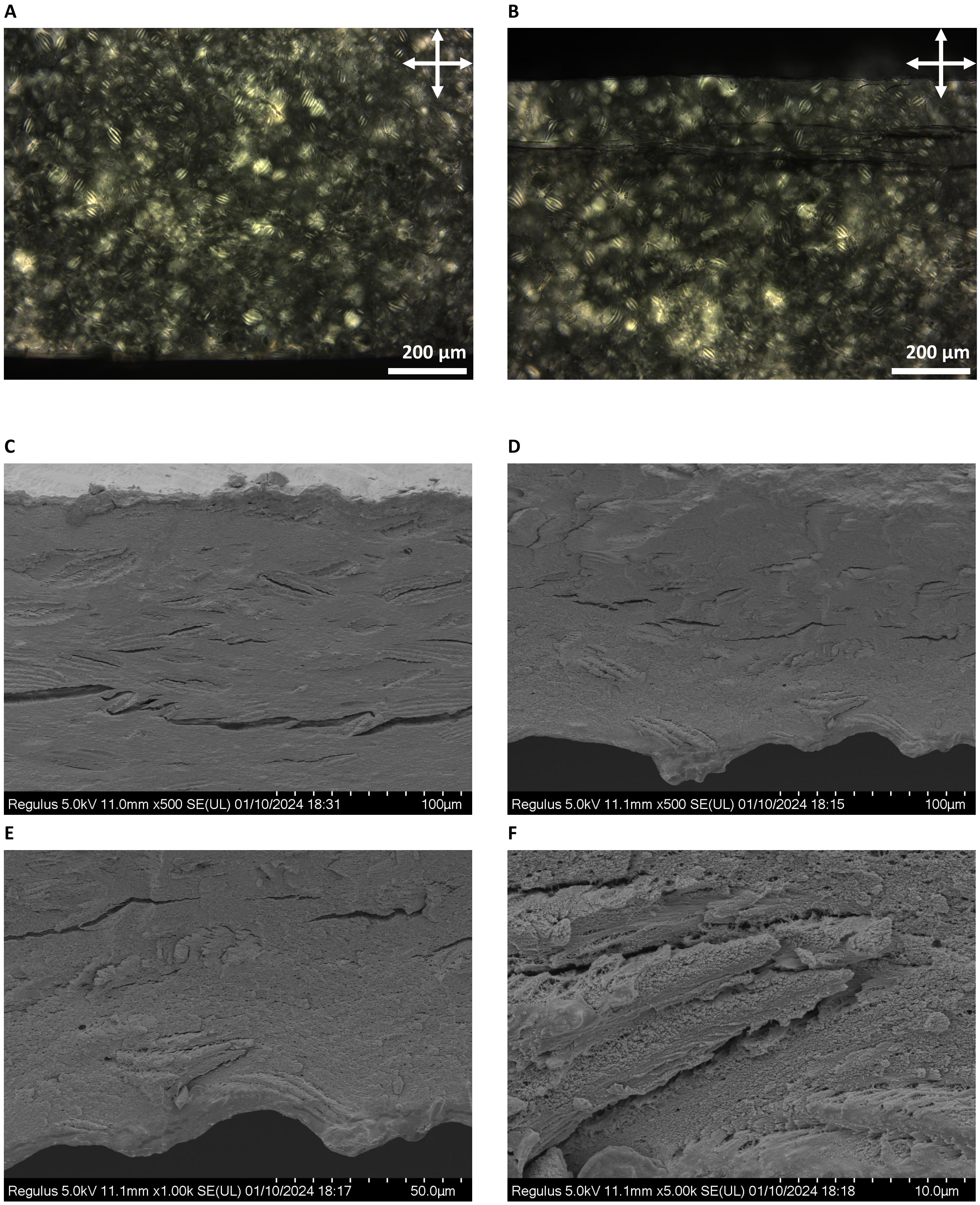

Figure S8. Cross-sectional polarized optical microscopy (**A**-**B**) and scanning electron microscopy (**C**-**F**) images of 1g_10minutes samples.

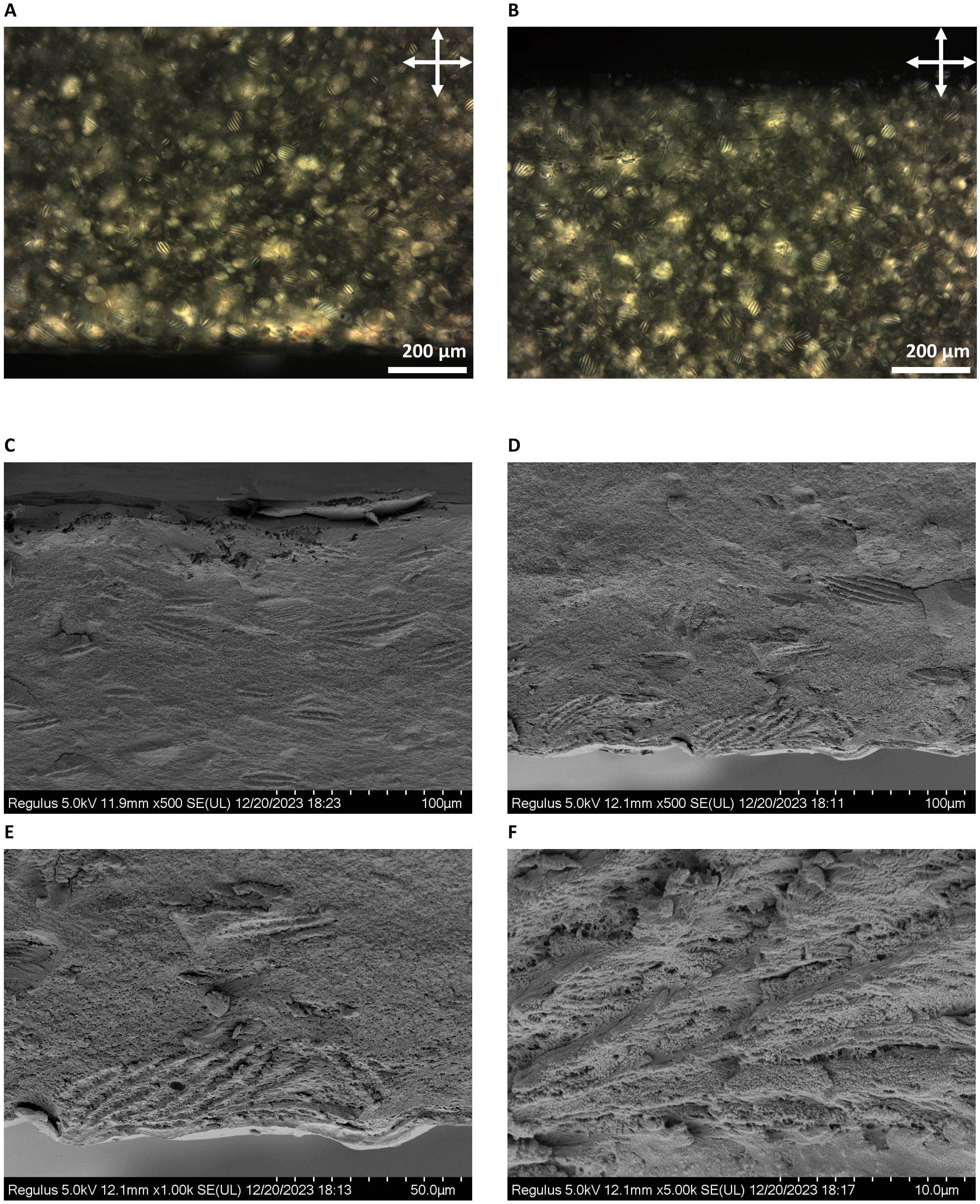

Figure S9. Cross-sectional polarized optical microscopy (**A**-**B**) and scanning electron microscopy (**C**-**F**) images of 1g_32minutes samples.

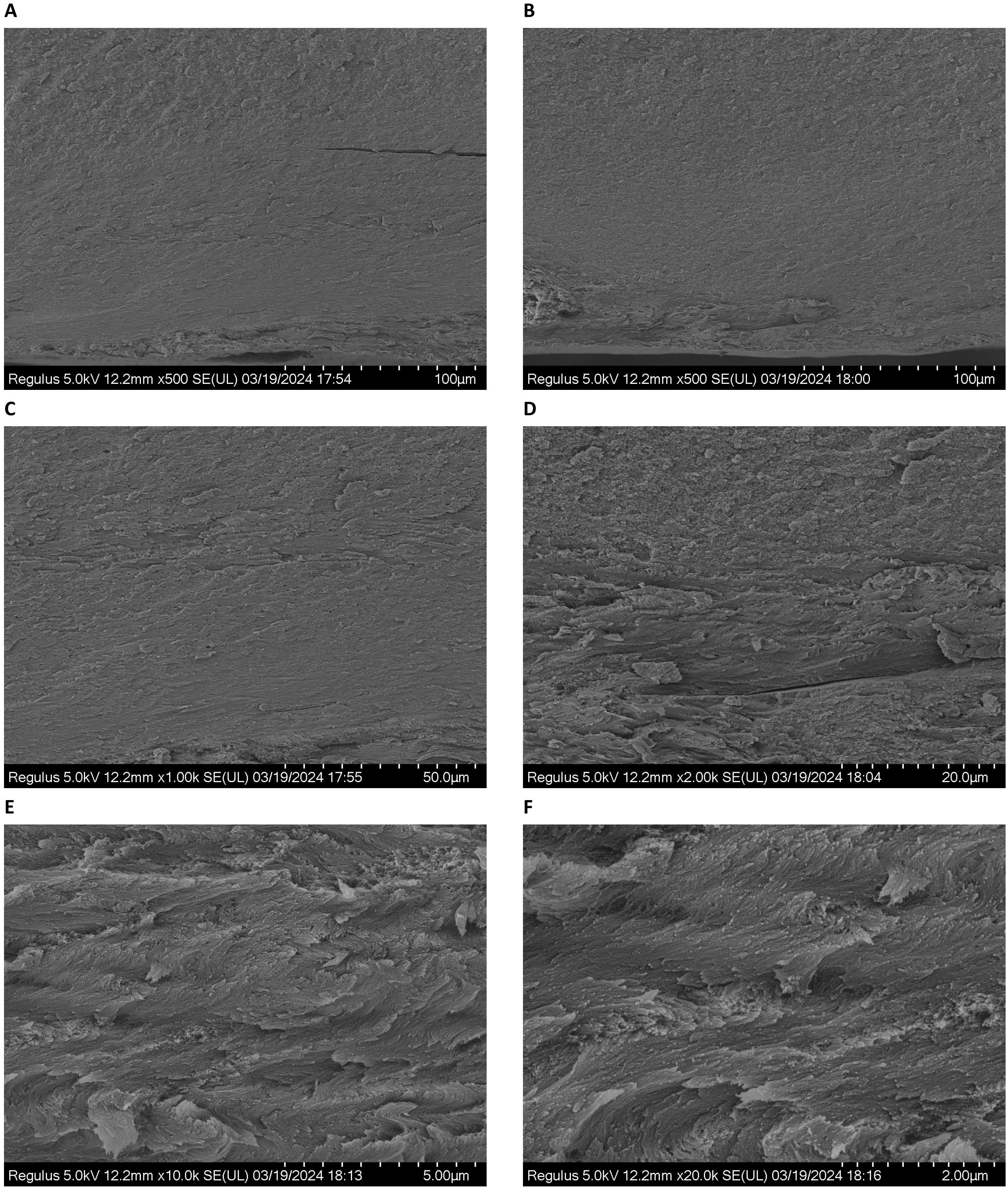

Figure S10. Cross-sectional scanning electron microscopy images of 7251g_10minutes samples.

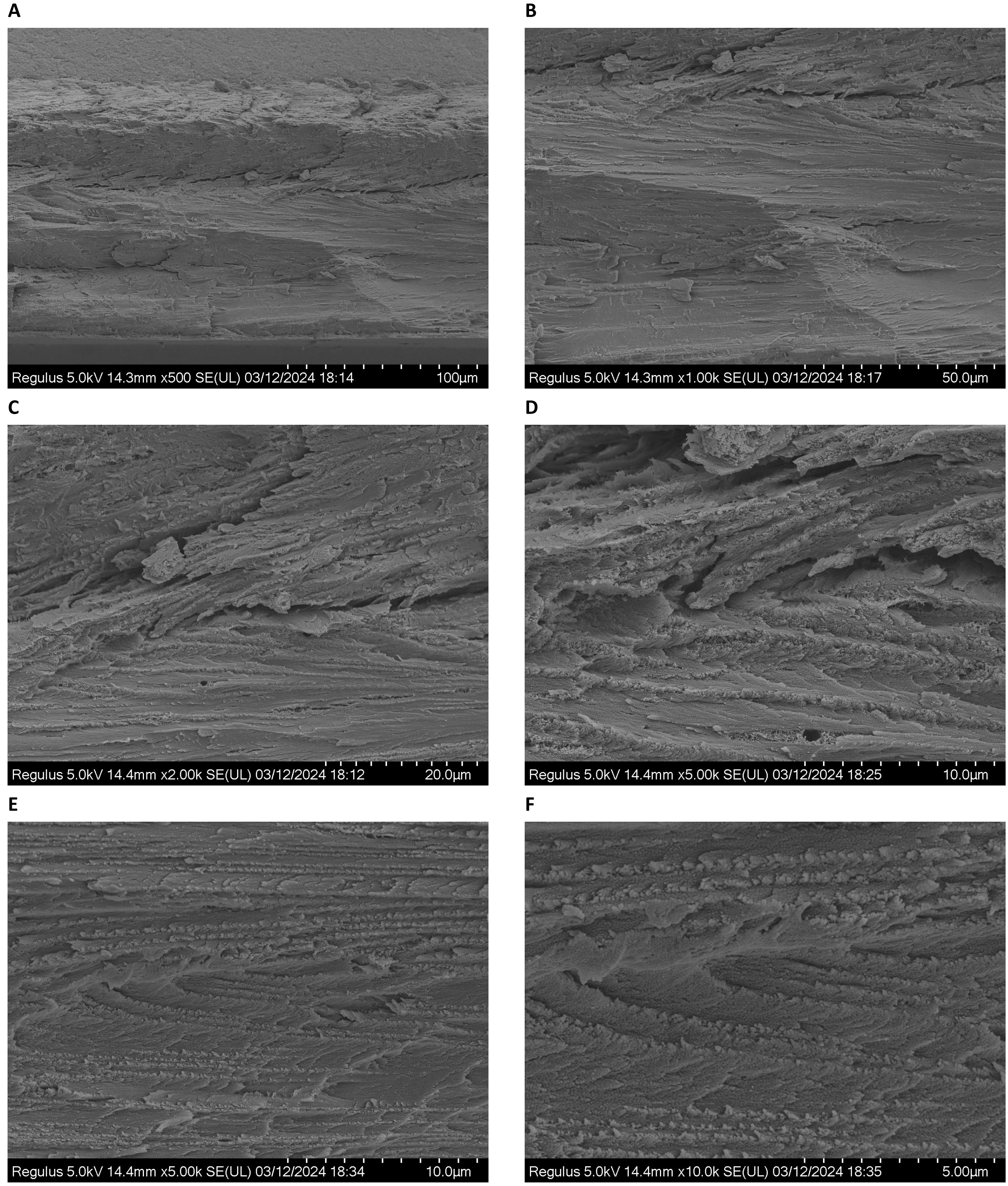

Figure S11. Cross-sectional scanning electron microscopy images of 7251g_32minutes samples.

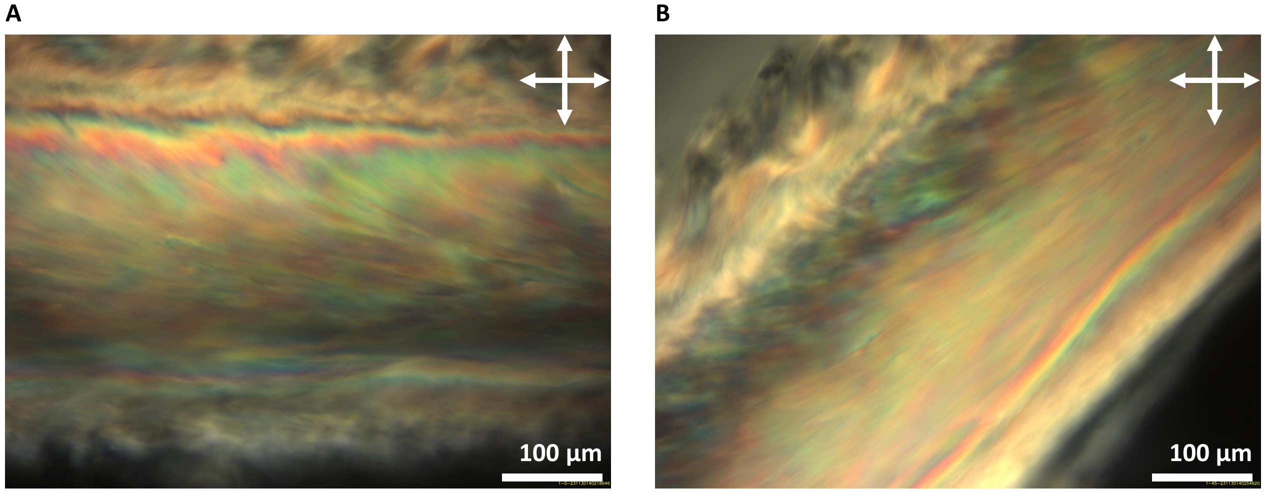

Figure S12. Polarized optical microscopy images (with two linear polarizers oriented in the North-South and East-West directions, illuminated by transmitted white light) of 7251g_62minutes samples.

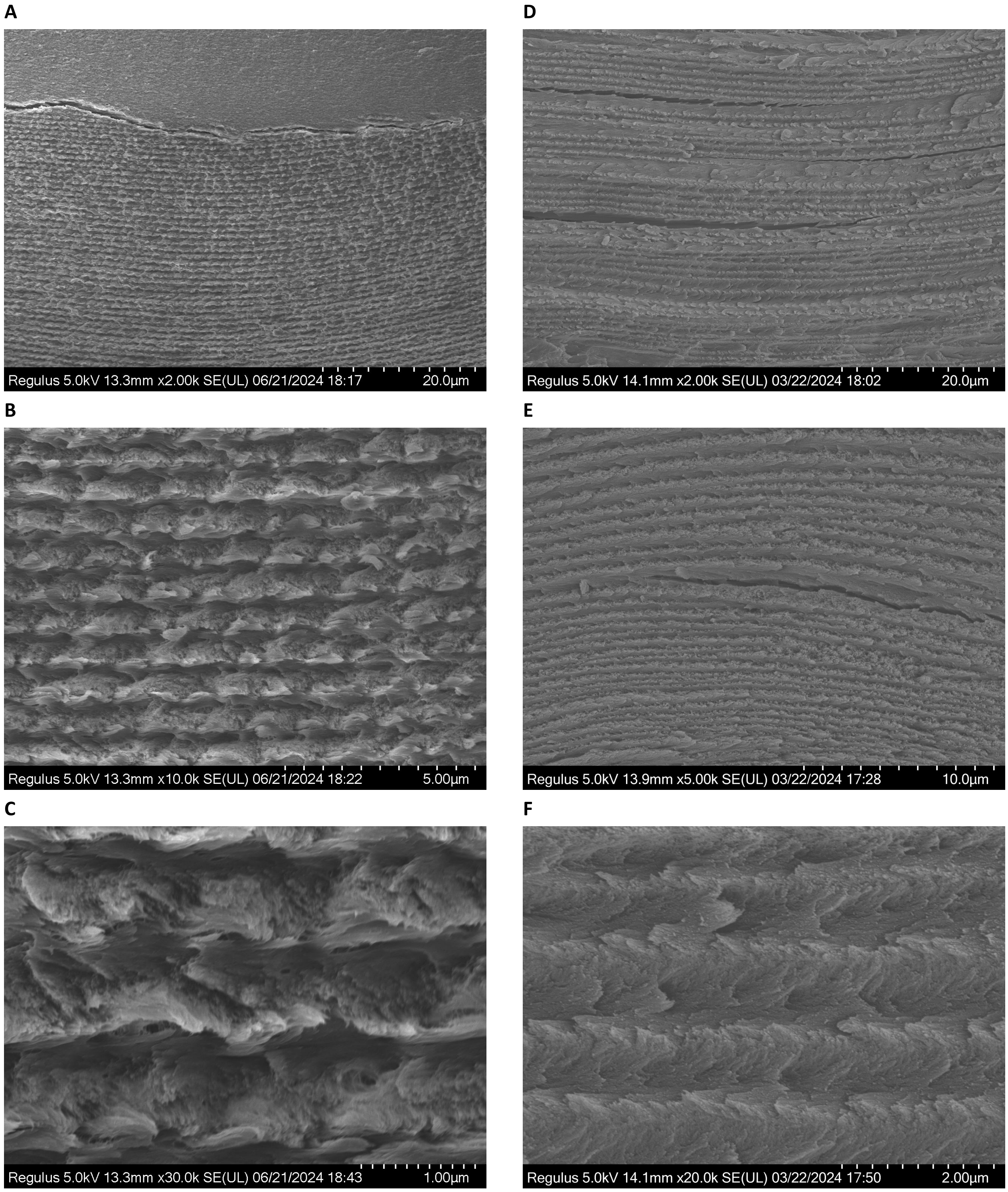

Figure S13. Cross-sectional scanning electron microscopy images of 7251g_62minutes samples.

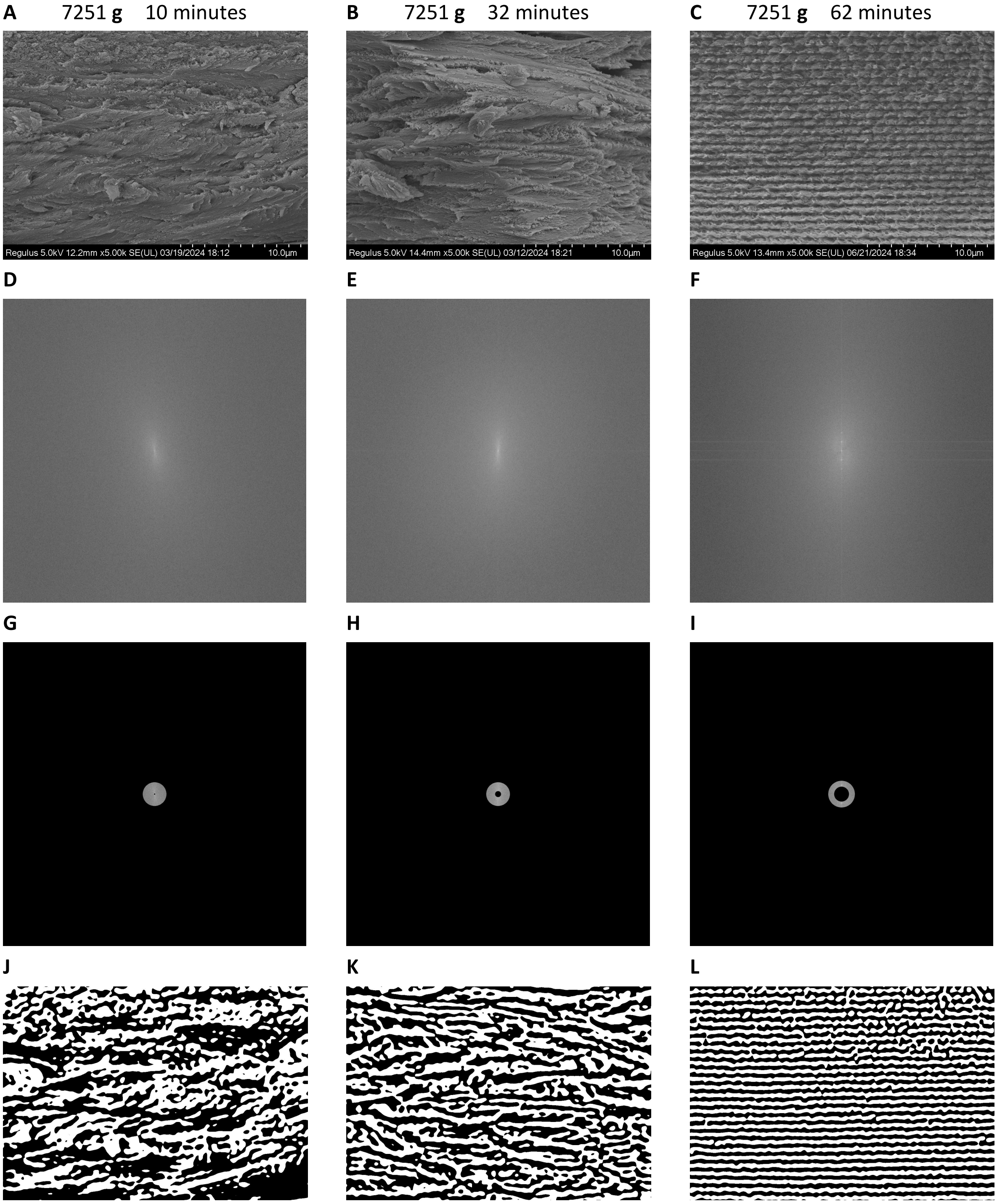

Figure S14. The original cross-sectional scanning electron microscopy images (**A**-**C**), two-dimensional discrete Fourier transformation (2D-DFT) results (**D**-**F**), extracted frequency domain regions (after applying band-pass filters) (**G**-**I**), and reconstructed spatial domain images (through two-dimensional inverse discrete Fourier transformations) showing the arrangements of chiral nematic layers (**J**-**L**) of 7251g_10minutes (the left column), 7251g_32minutes (the middle column), and 7251g_62minutes (the right column) samples.

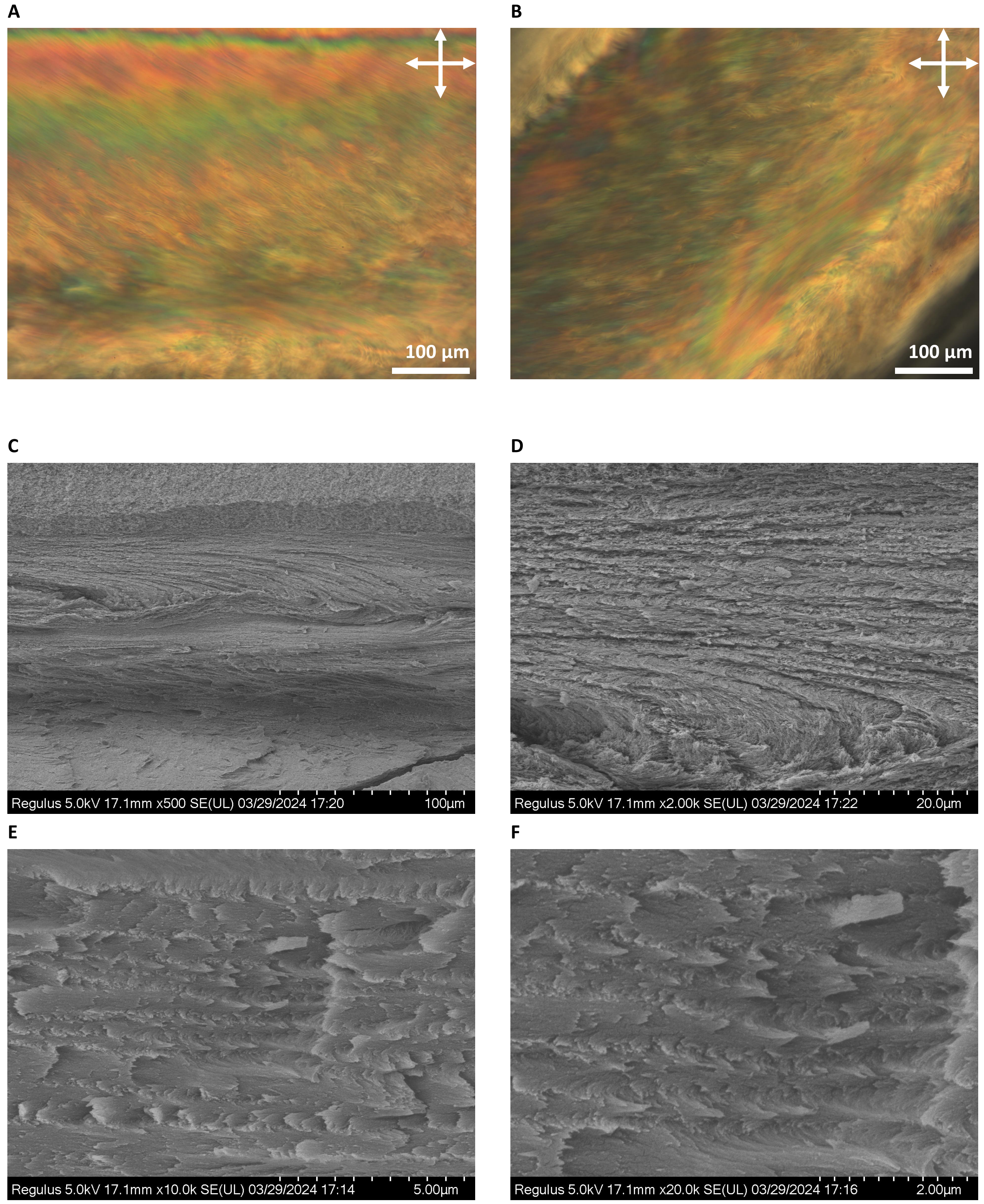

Figure S15. Cross-sectional polarized optical microscopy (**A**-**B**) and scanning electron microscopy (**C**-**F**) images of 3223g_62minutes samples.

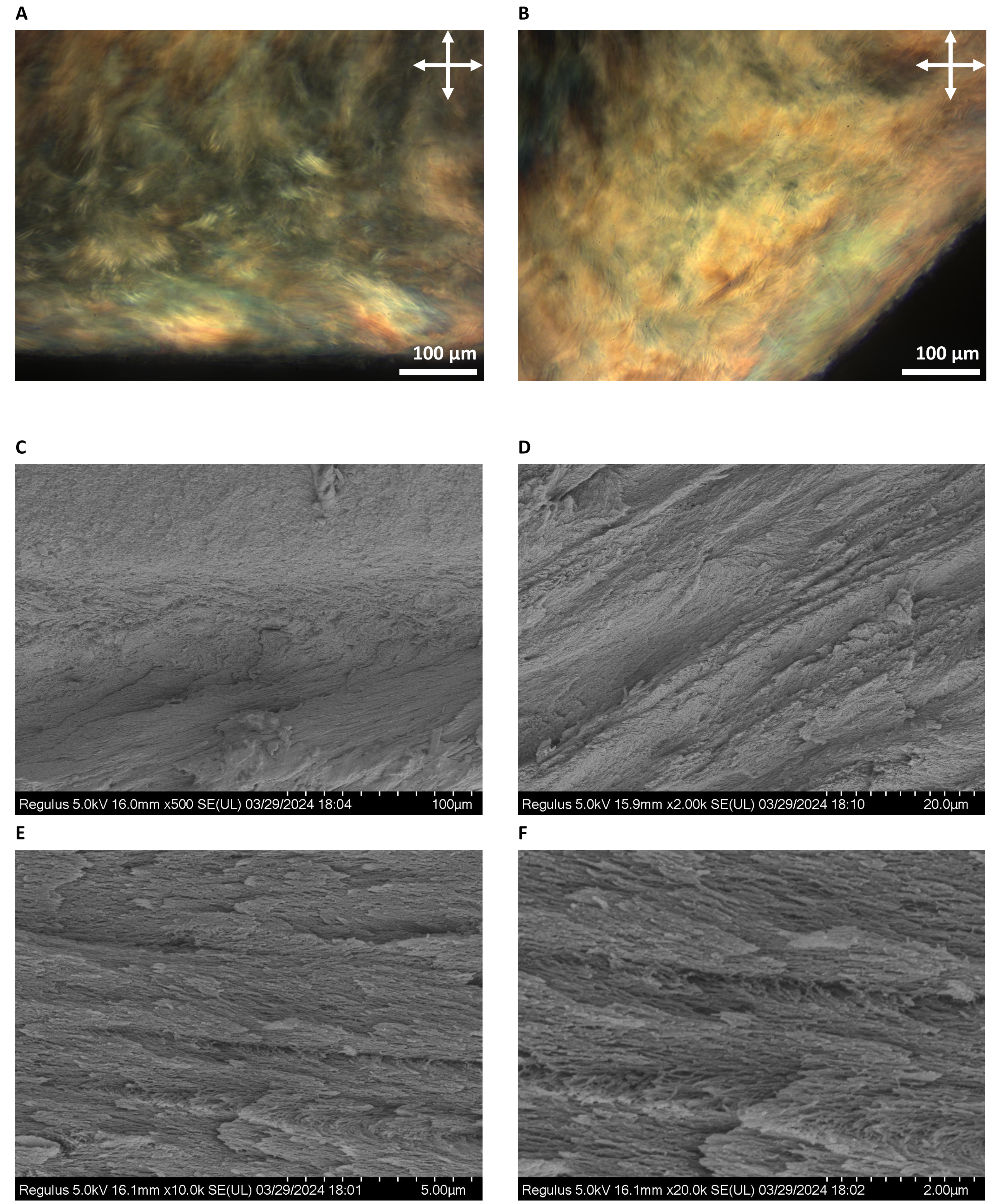

Figure S16. Cross-sectional polarized optical microscopy (**A**-**B**) and scanning electron microscopy (**C**-**F**) images of 806g_62minutes samples.

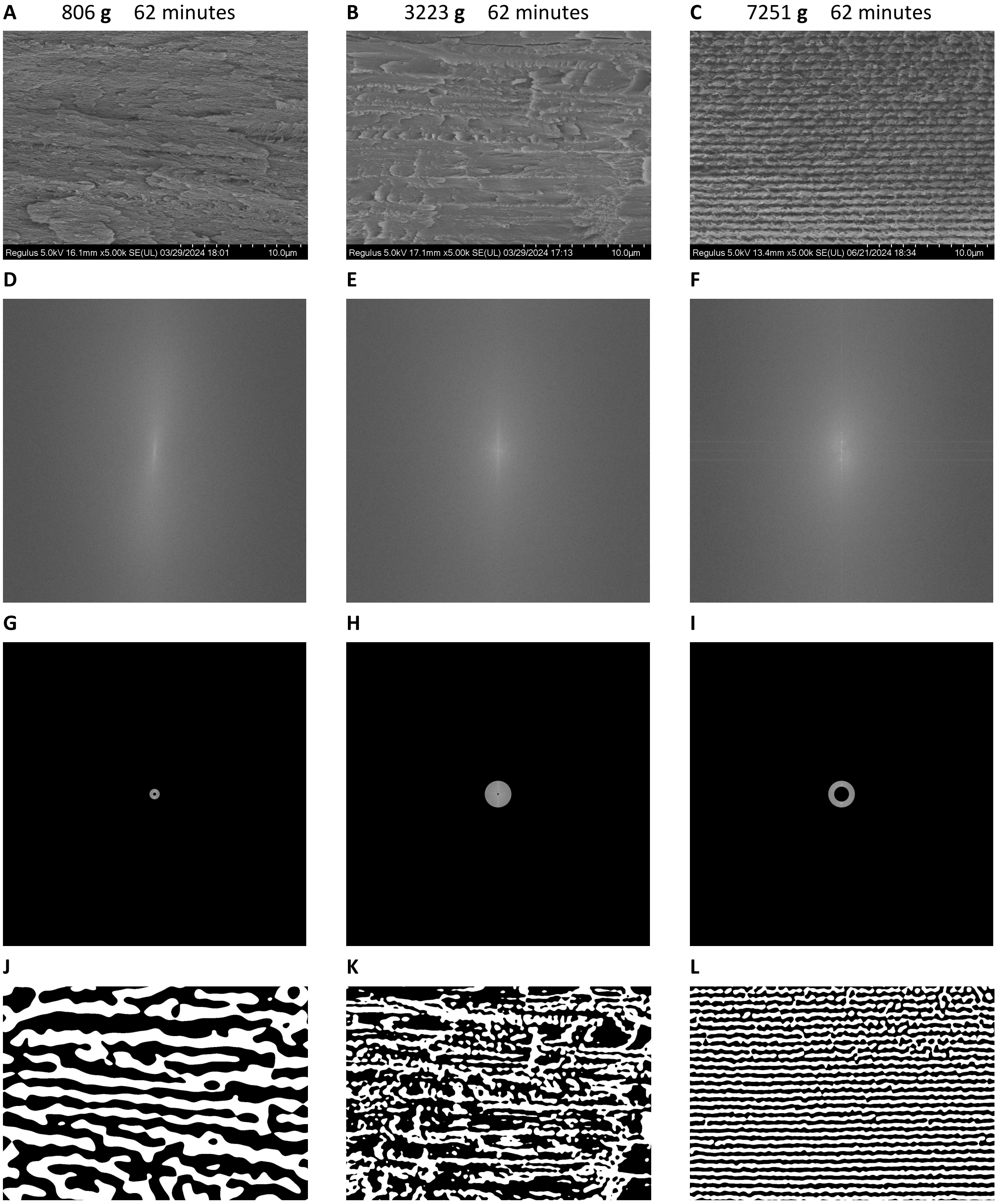

Figure S17. The original cross-sectional scanning electron microscopy images (**A**-**C**), two-dimensional discrete Fourier transformation (2D-DFT) results (**D**-**F**), extracted frequency domain components (after applying band-pass filters) (**G**-**I**), and reconstructed spatial domain images (through two-dimensional inverse discrete Fourier transformations) showing the arrangements of chiral nematic layers (**J**-**L**) of 806g_62minutes (the left column), 3223g_62minutes (the middle column), and 7251g_62minutes (the right column) samples.

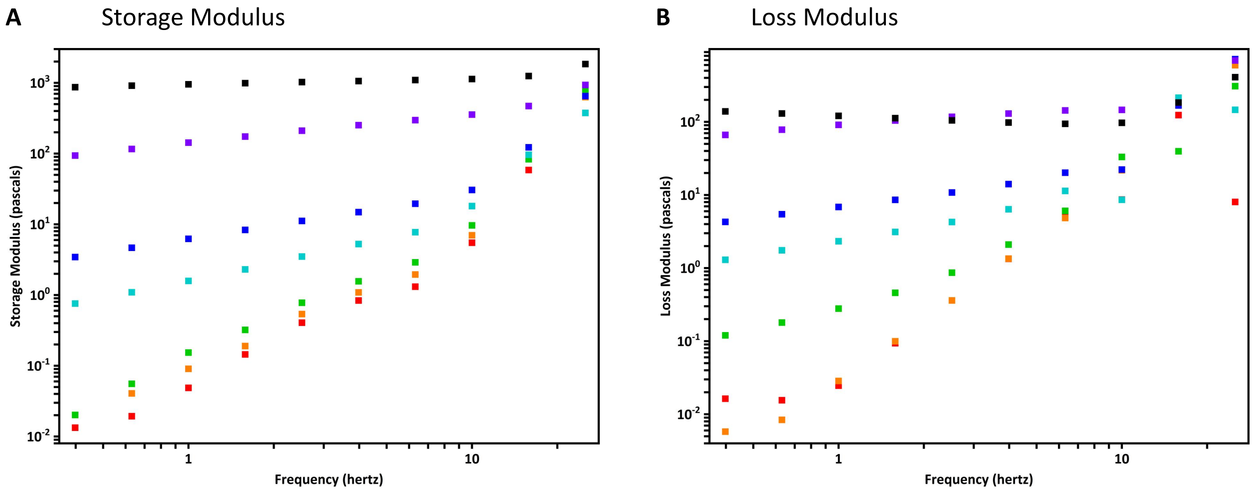

Figure S18. The storage modulus (**A**) and loss modulus (**B**) of 2.640 wt.% (red), 4.531 wt.% (orange), 5.625 wt.% (green), 7.336 wt.% (cyan), 7.971 wt.% (blue), 8.936 wt.% (violet), and 12.174 wt.% (black) cellulose nanocrystal dispersions measured in relation to the frequency.

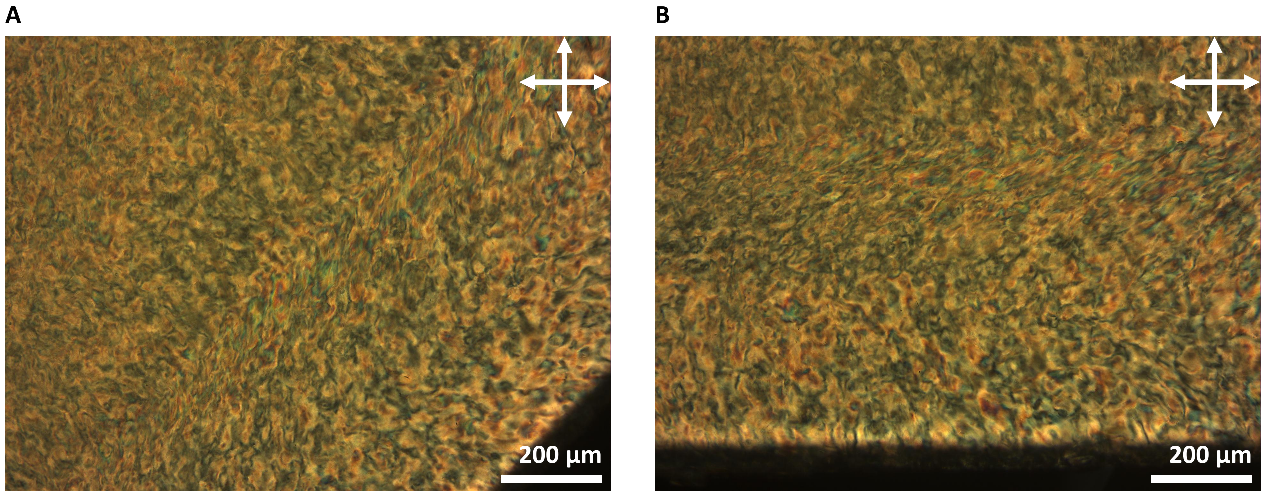

Figure S19. Cross-sectional polarized optical microscopy images showing the existence of topological defects in a 1g_62minutes_10wt% sample. From (**A**) to (**B**), the hydrogel cross section was rotated by 45 degrees.

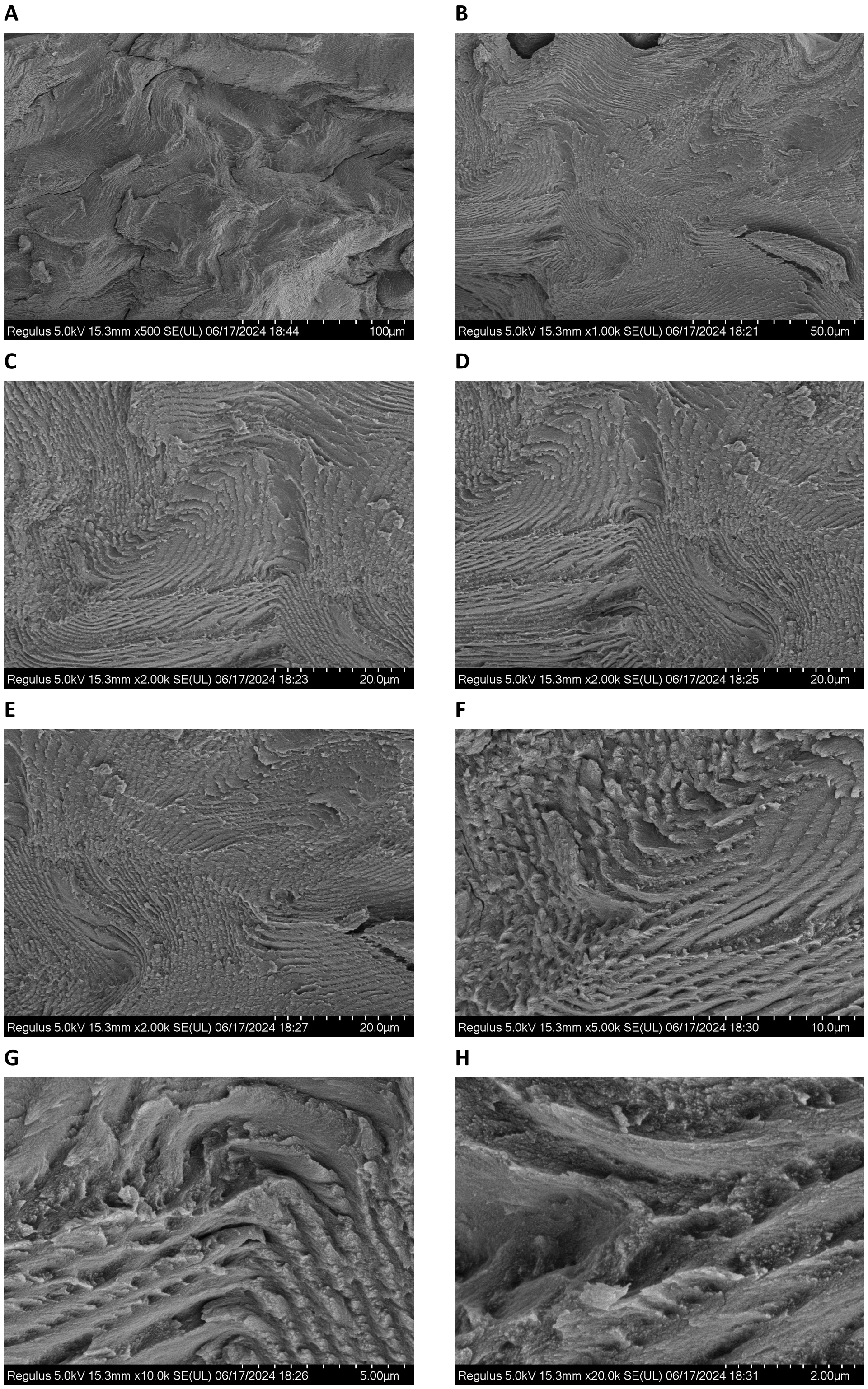

Figure S20. Cross-sectional scanning electron microscopy images of 1g_62minutes_10wt% samples.

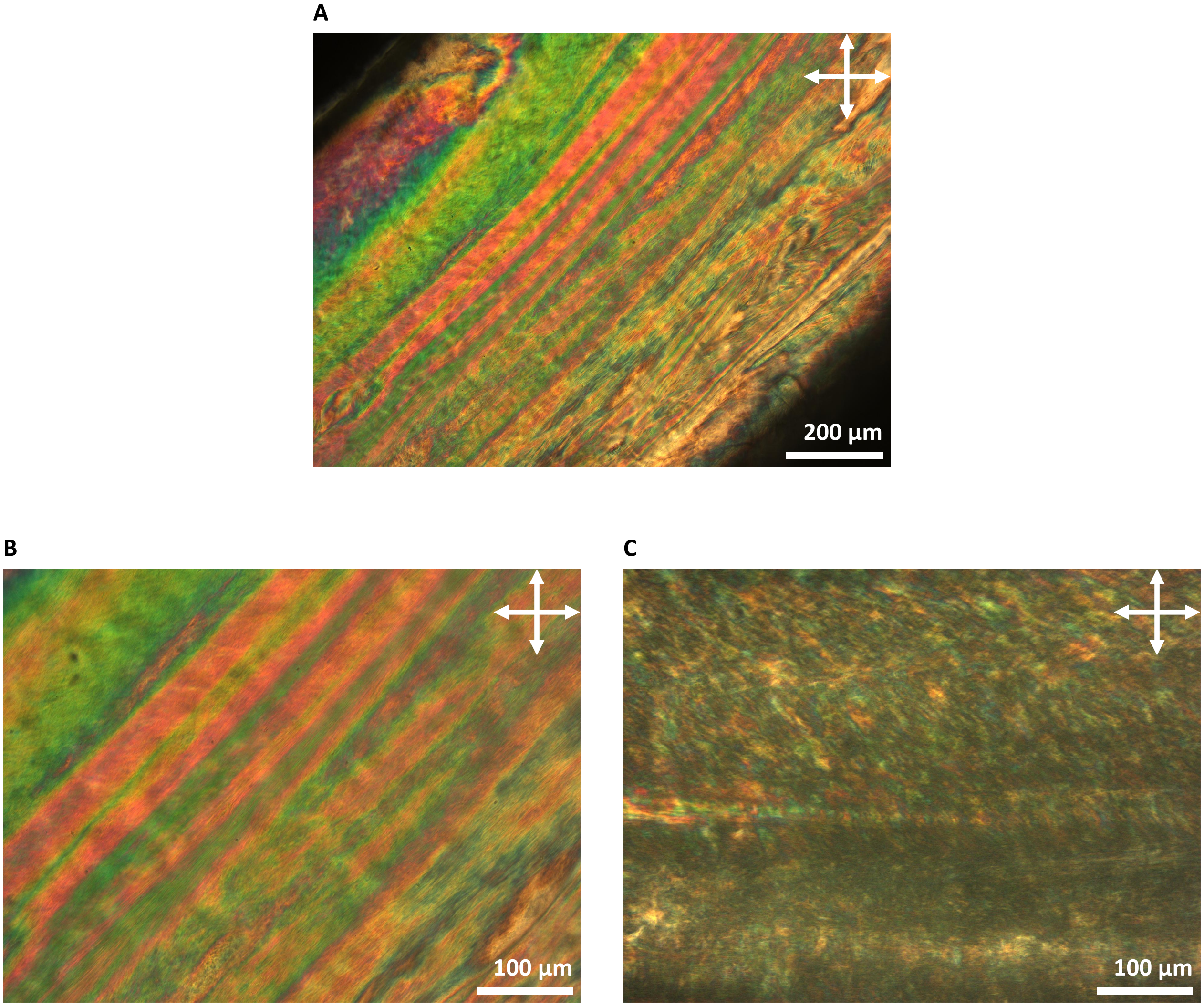

Figure S21. Cross-sectional polarized optical microscopy images of a 7251g_62minutes_10wt% sample. From (**B**) to (**C**), the hydrogel cross section was rotated by 45 degrees.

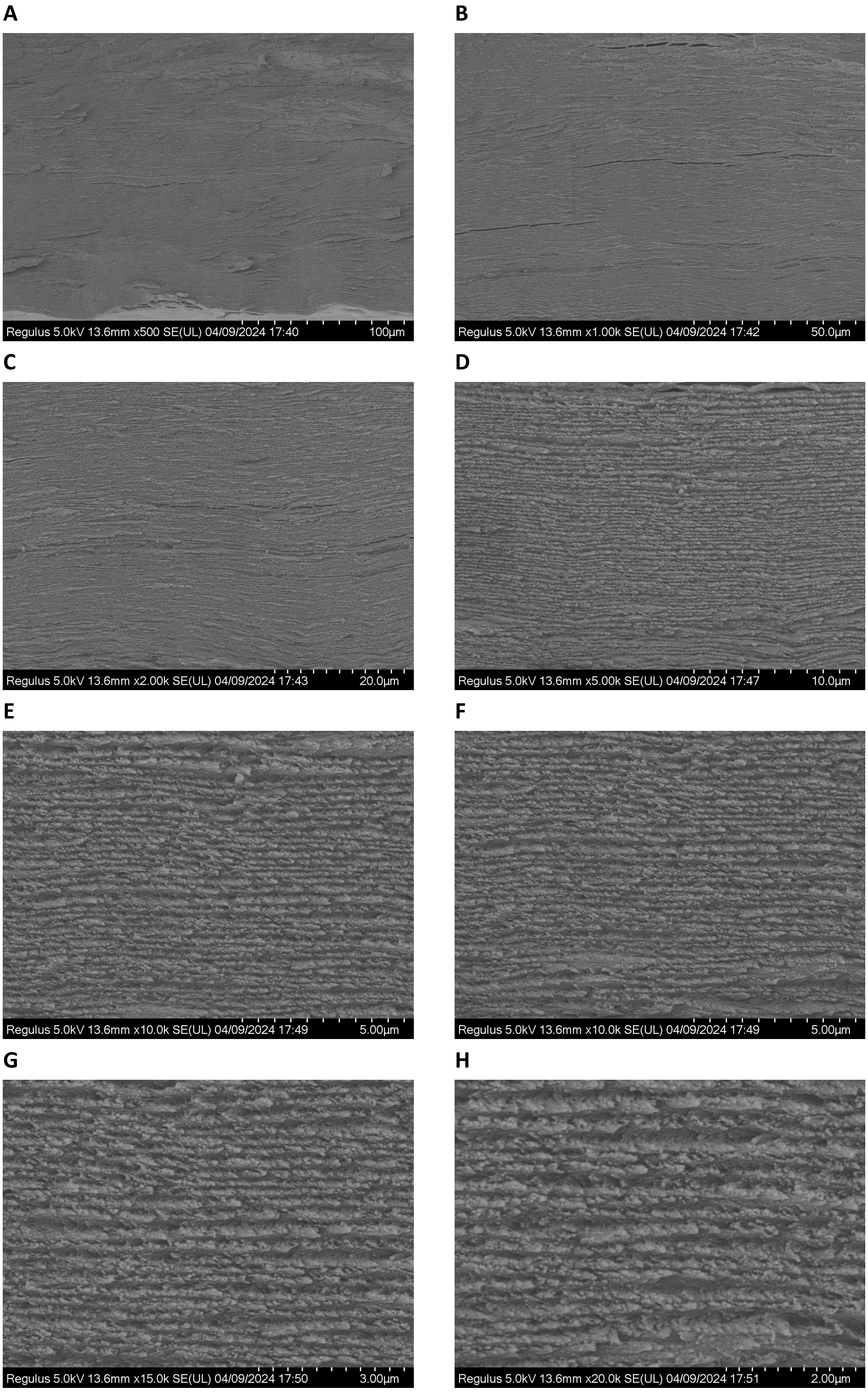

Figure S22. Cross-sectional scanning electron microscopy images of 7251g_62minutes_10wt% samples.

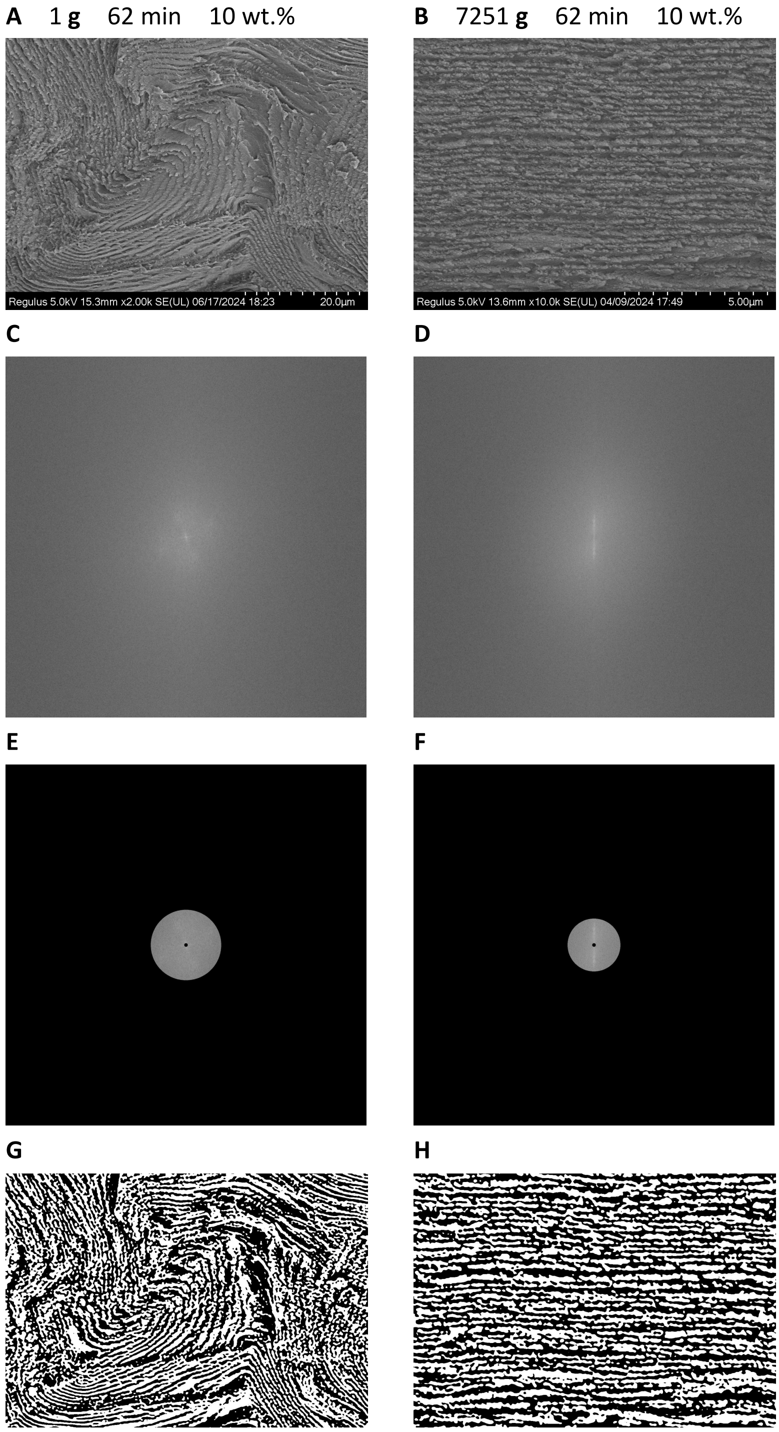

Figure S23. The original cross-sectional scanning electron microscopy images (**A**-**B**), two-dimensional discrete Fourier transformation (2D-DFT) results (**C**-**D**), extracted frequency domain components (after applying band-pass filters) (**E**-**F**), and reconstructed spatial domain images (by two-dimensional inverse discrete Fourier transformations) showing the arrangements of chiral nematic layers (**G**-**H**) of 1g_62minutes_10wt% (the left column) and 7251g_62minutes_10wt% (the right column) samples.

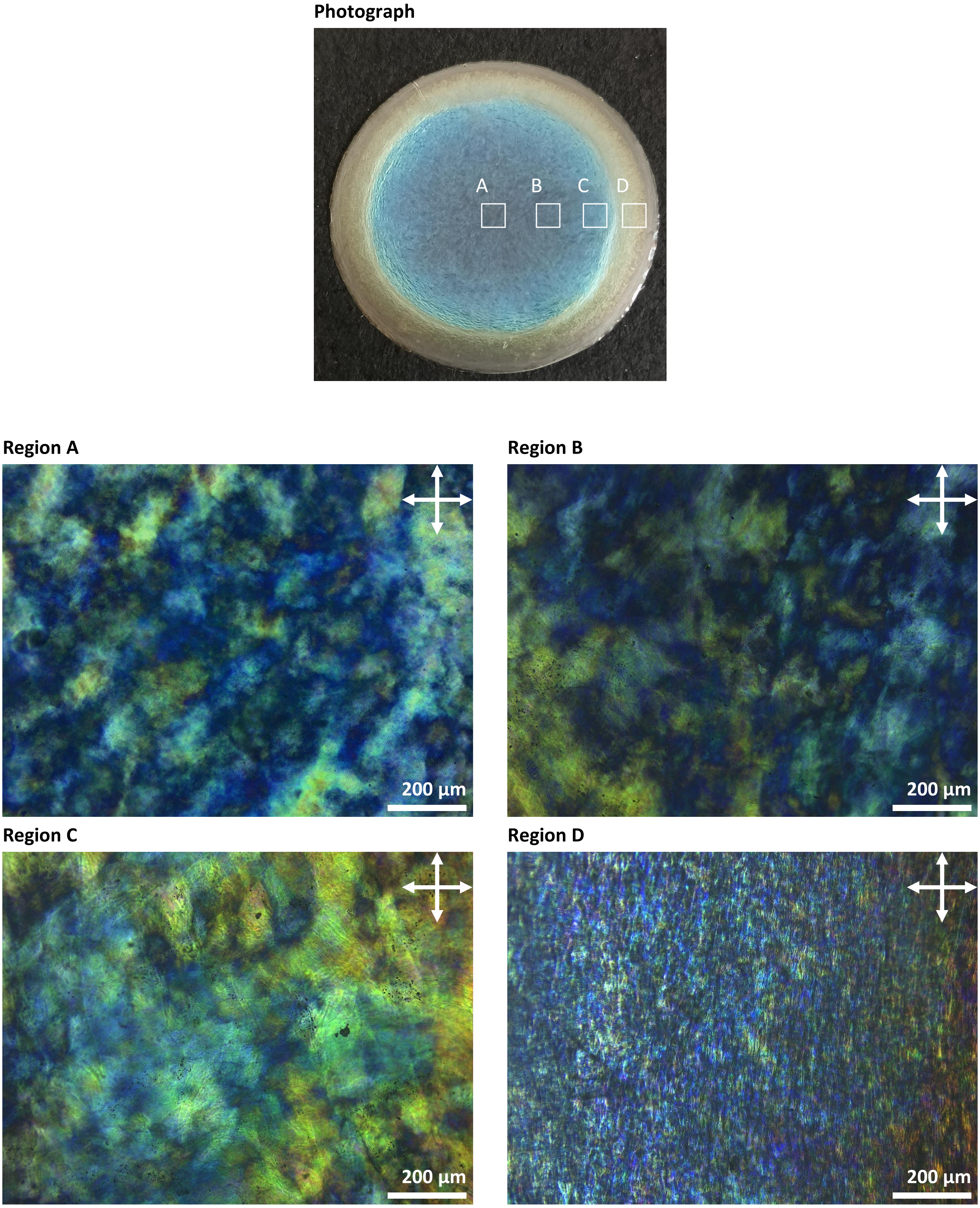

Figure S24. Polarized optical microscopy images taken from different regions of a dried 1g_62min_10wt% film.

Table S1. Shrinkage of Cylindrical-Shaped CNC/Polyacrylamide Composite Hydrogels During Drying.

	Average Diameter (mm)	Average Thickness (mm)	Concentration of Cellulose Nanocrystals
Freshly-Prepared Hydrogel	19.24	5.27	5.6 wt.%
Completely Dried Hydrogel	14.37	2.35	
Degree of Shrinkage	25.31%	55.41%	
Freshly-Prepared Hydrogel	19.42	4.53	10.1 wt.%
Completely Dried Hydrogel	14.54	2.01	
Degree of Shrinkage	25.13%	55.63%	
